# Supplementary material for: Forecasting coal power plant retirement ages and lock-in with random forest regression
Source: Patterns (N Y). 2023 Jun 21;4(7):100776. doi: 10.1016/j.patter.2023.100776 (PMC10382988; doi:10.1016/j.patter.2023.100776)
Supplement: Document S1. Figure S1, Tables S1–S10, and Note S1 [file mmc1.pdf]

Patterns, Volume *4*

Supplemental information

Forecasting coal power plant retirement ages and lock-in with random forest regression

Achmed Edianto, Gregory Trencher, Niccolò Manych, and Kazuyo Matsubae

Supplemental Information for

Forecasting coal power plant retirement ages and lock-in with random forest regression

Achmed Edianto, Gregory Trencher, Niccolò Manych, Kazuyo Matsubae

Supplemental graph for global coal-fired power plant trends

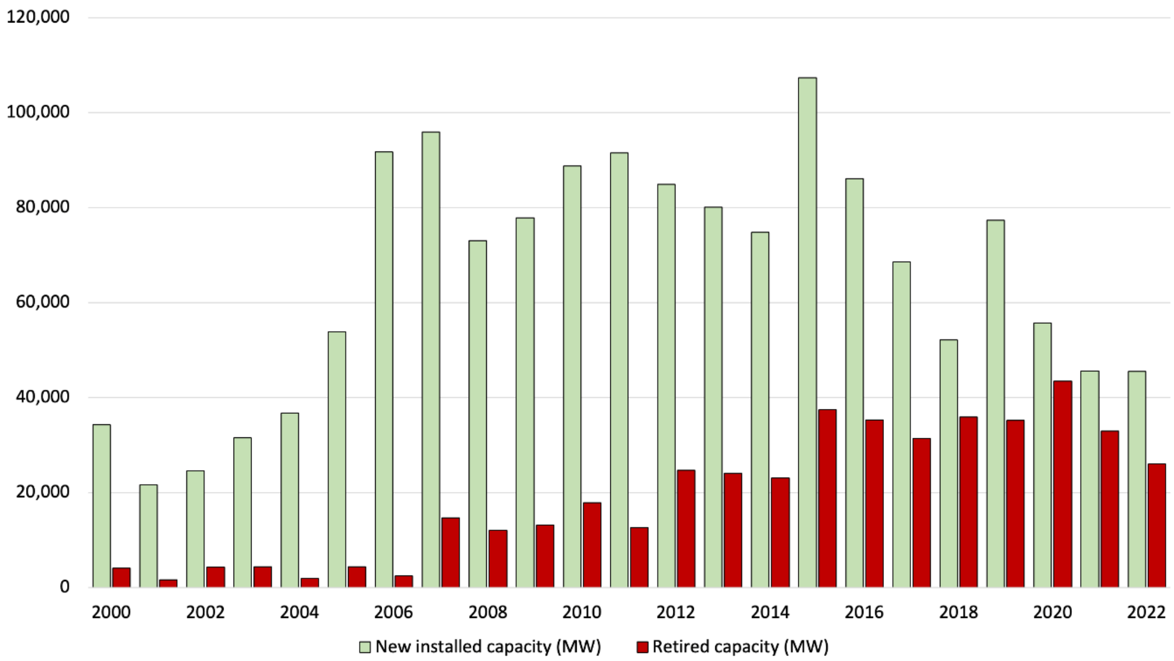

Figure S1. New installed capacity (MW) vs retired capacity (MW) (Global Energy Monitor 2022)

Table S1. Status of coal-fired power plants by country (as of 2022)

Country	Announced + Pre-permit + Permitted		Construction		Operating		Retired 2000-2022	
	MW	Unit	MW	Unit	MW	Unit	MW	Unit
Albania	0	0	0	0	0	0	0	0
Argentina	0	0	120	1	495	2	0	0
Australia	1,000	1	0	0	23,977	57	7,107	50
Austria	0	0	0	0	0	0	1,993	10
Bangladesh	6,700	7	5,724	9	2,855	7	0	0
Belarus	0	0	0	0	0	0	0	0
Belgium	0	0	0	0	0	0	2,865	19
Bosnia and Herzegovina	1,350	4	0	0	2,073	10	0	0
Botswana	450	2	0	0	732	8	0	0
Brazil	1,666	5	0	0	3,177	17	518	5
Brunei	0	0	0	0	220	4	0	0
Bulgaria	0	0	0	0	4,709	30	1,380	8
Cambodia	700	2	315	3	1,405	9	0	0
Canada	0	0	0	0	4,707	19	14,818	47
Chile	0	0	0	0	4,323	20	1,193	8
China	250,069	400	115,472	211	1,092,889	3,092	119,268	1,087
Colombia	0	0	0	0	1,646	14	0	0
Côte d'Ivoire	0	0	0	0	0	0	0	0
Croatia	0	0	0	0	210	1	0	0
Czech Republic	0	0	0	0	7,445	69	2,997	18
Denmark	0	0	0	0	1,560	4	3,443	14
Djibouti	0	0	0	0	0	0	0	0
Dominican Republic	0	0	0	0	1,064	5	0	0
DR Congo	0	0	0	0	0	0	0	0
Egypt	0	0	0	0	0	0	0	0
El Salvador	0	0	0	0	0	0	0	0
Eswatini	300	3	0	0	0	0	0	0
Ethiopia	0	0	0	0	0	0	0	0
Finland	0	0	0	0	1,468	8	2,319	15
France	0	0	0	0	2,507	11	6,619	23
Georgia	0	0	0	0	0	0	0	0
Germany	0	0	0	0	40,505	102	25,181	122
Ghana	0	0	0	0	0	0	0	0
Greece	0	0	660	1	2,225	7	3,053	14
Guadeloupe	0	0	0	0	64	2	38	1
Guatemala	0	0	0	0	1,172	16	0	0
Guinea	0	0	0	0	0	0	0	0
Honduras	0	0	0	0	105	3	0	0
Hong Kong	0	0	0	0	6,110	14	500	2
Hungary	0	0	0	0	944	7	515	10

India	28,503	44	32,000	49	234,256	840	15,651	158
Indonesia	7,480	30	18,849	60	40,647	231	0	0
Iran	0	0	650	2	0	0	0	0
Ireland	0	0	0	0	915	3	0	0
Israel	0	0	0	0	4,325	9	575	1
Italy	0	0	0	0	6,166	14	4,194	21
Jamaica	0	0	0	0	0	0	0	0
Japan	500	1	2,450	4	52,978	152	2,674	19
Kazakhstan	756	2	65	1	13,033	95	210	4
Kenya	64	1	0	0	0	0	0	0
Kosovo	0	0	0	0	1,290	5	190	2
Kyrgyzstan	600	1	0	0	826	9	195	3
Laos	7,036	12	0	0	1,878	3	0	0
Latvia	0	0	0	0	0	0	0	0
Madagascar	30	1	0	0	120	3	0	0
Malawi	400	7	0	0	0	0	0	0
Malaysia	0	0	0	0	13,280	25	0	0
Mauritius	0	0	0	0	195	5	0	0
Mexico	0	0	0	0	5,378	15	0	0
Moldova	0	0	0	0	0	0	0	0
Mongolia	7,030	10	50	1	960	11	0	0
Montenegro	0	0	0	0	225	1	0	0
Morocco	0	0	0	0	4,257	14	0	0
Mozambique	1,200	6	0	0	0	0	0	0
Myanmar	0	0	0	0	190	4	0	0
Namibia	0	0	0	0	120	4	0	0
Netherlands	0	0	0	0	4,152	5	3,665	6
New Zealand	0	0	0	0	500	2	500	2
Niger	200	4	0	0	0	0	0	0
Nigeria	0	0	0	0	285	7	0	0
North Korea	0	0	0	0	3,250	45	0	0
North Macedonia	0	0	0	0	800	4	0	0
Oman	0	0	0	0	0	0	0	0
Pakistan	4,010	8	732	3	7,638	20	0	0
Panama	0	0	0	0	306	2	120	3
Papua New Guinea	0	0	0	0	0	0	0	0
Peru	0	0	0	0	0	0	135	1
Philippines	1,620	9	735	5	11,893	58	157	3
Poland	0	0	100	1	29,130	145	7,124	60
Portugal	0	0	0	0	0	0	2,028	7
Romania	0	0	0	0	2,955	14	4,450	34
Russia	3,173	12	70	2	39,925	312	8,301	62
Senegal	0	0	0	0	155	2	0	0
Serbia	1,350	4	350	1	4,405	17	32	1
Slovakia	0	0	0	0	769	7	724	8

Slovenia	0	0	0	0	1,069	5	535	5
South Africa	1,635	2	1,600	2	43,624	90	1,180	15
South Korea	0	0	3,140	3	39,154	80	3,420	11
Spain	0	0	0	0	2,210	8	10,892	40
Sri Lanka	0	0	0	0	900	3	0	0
Sudan	0	0	0	0	0	0	0	0
Sweden	0	0	0	0	0	0	291	3
Syria	0	0	0	0	0	0	0	0
Taiwan	0	0	0	0	19,244	55	2,234	14
Tajikistan	0	0	0	0	400	4	0	0
Tanzania	600	4	0	0	0	0	0	0
Thailand	600	1	0	0	6,138	21	675	6
Türkiye	10,438	22	145	1	20,093	77	360	1
Ukraine	660	2	0	0	9,320	56	3,821	13
United Arab Emirates	0	0	0	0	0	0	1,200	2
United Kingdom	0	0	0	0	4,140	8	30,623	82
United States	0	0	0	0	212,042	441	148,514	738
Uzbekistan	600	2	0	0	2,493	12	241	4
Venezuela	0	0	0	0	0	0	0	0
Vietnam	1,210	6	6,120	10	24,637	73	0	0
Zambia	300	2	0	0	330	3	0	0
Zimbabwe	4,570	17	940	4	1,000	8	0	0
**Total**	346,800	634	190,287	374	2,082,581	6,565	448,718	2,782

Note S1. Procedure for literature review

Consisting of three segments, the string essentially sets our core topic (coal power, coal plant, etc.), the act of retirement or construction (closure, shutdown, development etc.) and influencing conditions (influence, driver etc.). This was iteratively developed through a procedure where we set search terms, verified the accuracy and coverage of hits, identified other important search terms from results and then, finally, fine-tuned the search string until an acceptable level was achieved.

The search was carried out on October 2021 with the following conditions:

- Publication type: Articles and reviews
- Publication language: English
- Search scope: Title, author keywords, abstract
- Temporal scope: All papers published up to October 31, 2021
- Excluded academic fields: Agriculture, biochemical, chemical, chemical engineering, computer science, earth science, engineering, immunology, mathematic, medicine, material science, neuroscience, nursing, pharmacy, physics.

The search string, in Scopus language, appears as follows. We set wild cards (*) to ensure that synonyms for CFPP closure and retirement were found independently from word endings:

TITLE-ABS-KEY ("coal power" OR "coal generation" OR "coal electricity" OR "coal-fired" OR "coal unit" OR "coal plant") AND TITLE-ABS-KEY ("clos*" OR "closure" OR "phase-out" OR "phasing-out" OR "shut-down" OR "retir*" OR "construct" OR "develop" OR "build" OR "decline" OR "stranded" OR "stranding") AND TITLE-ABS-KEY ("factor" OR "influence" OR "indicator" OR "condition" OR "driver" OR "barrier") AND DOCTYPE (ar OR re) AND

PUBYEAR > 2000 AND (EXCLUDE (SUBJAREA , "ENGI") OR EXCLUDE (SUBJAREA , "CENG") OR EXCLUDE (SUBJAREA , "CHEM") OR EXCLUDE (SUBJAREA , "EART") OR EXCLUDE (SUBJAREA , "MATE") OR EXCLUDE (SUBJAREA , "PHYS") OR EXCLUDE (SUBJAREA , "MATH") OR EXCLUDE (SUBJAREA , "MEDI") OR EXCLUDE (SUBJAREA , "COMP") OR EXCLUDE (SUBJAREA , "AGRI") OR EXCLUDE (SUBJAREA , "BIOC") OR EXCLUDE (SUBJAREA , "PHAR") OR EXCLUDE (SUBJAREA , "NEUR") OR EXCLUDE (SUBJAREA , "IMMU") OR EXCLUDE (SUBJAREA , "NURS")) AND (LIMIT-TO (LANGUAGE , "English"))

The above search procedure resulted in 194 potentially relevant publications to which we added further ones identified from the in-text citations, during the later stages of our review. To identify relevant publications, we set up criteria to be met by evidence contained in the title and abstract. That is, we only considered studies that:

- Explicitly mention drivers or barriers that affect coal power development, retirement or phase-out
- Explicitly analyze the influence of these factors on coal power development, retirement or phase-out

Application of these criteria led to a set of 25 potentially relevant publications. We then read the full-text versions and further excluded those deemed irrelevant. We also excluded publications for which the full text was unavailable. This procedure resulted in a final corpus of 19 publications that were subsequently examined (**Table 2 in main text**).

Data

Table S2. Sample description

	**2000-2009**		**2010-2020**	
	Units	MW	Units	MW
	357	31,872	626	80,824
**United States**	84	8,727	589	117,821
**Germany**	28	3,358	114	11,326
**United Kingdom**	28	4,342	75	15,073
**India**	20	1,881	52	25,187
**Canada**	13	2,718	42	6,011
**Belgium**	9	998	41	6,597
**Japan**	9	1,417	39	4,827
**Poland**	8	712	30	7,704
**Others**	55	6,683	229	46,298
**Total**	611	62,708	1,837	321,667

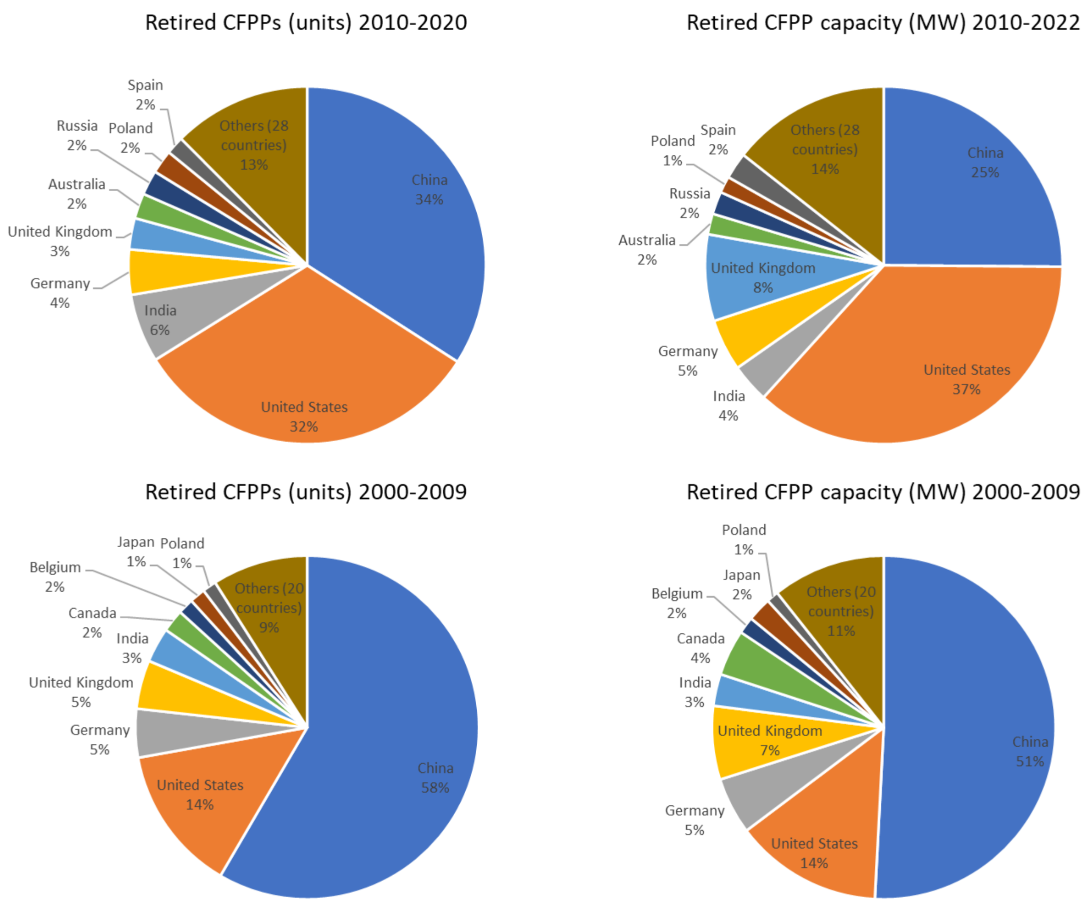

Figure S2. Distribution of retired CFPPs in historical analysis

Table S3. Retirement reasons of the outlier units (retired between 3-9 years after the start of commercial operation)

**Global Energy Monitor wiki page**	**Country**	**Subnational (province, state)**	**Unit name**	**Plant name**	**Capacity (MW)**	**Year of starting operation**	**Year of retirement**	**Retirement age (years)**	**Notes (taken from Global Energy Monitor wiki page)**
https://gem.wiki/Callide_Oxyfuel_Project	Australia	Queensland	Callide Oxyfuel Project	Callide Oxyfuel Project	30	2012	2015	3	This unit was recommissioned for a carbon capture and storage pilot project for only 3 years.
https://gem.wiki/Lianzhou_power_station	China	Guangdong	Yuelian power station Unit 3	Yuelian power station	135	2004	2013	9	The full plant was retired in 2013, after a breakout of high levels of lead in the blood of the local children.
https://gem.wiki/Lianzhou_power_station	China	Guangdong	Yuelian power station Unit 4	Yuelian power station	135	2004	2013	9	Same as above
https://gem.wiki/Guiyang_power_station	China	Guizhou	Guiyang power station Unit 4	Guiyang power station	200	2004	2013	9	This unit is part of the power plant that was founded in 1957. Since the whole power plant retired 2013, this unit need to retired although it is just operating for 9 years.
https://gem.wiki/Huadian_Shijiazhuang_power_station	China	Hebei	Huadian Shijiazhuang power station Unit 16	Huadian Shijiazhuang power station	125	2005	2014	9	After the plant was blamed for its contribution to the city's severe air pollution, this plant was forced by government authorities to retire some units, including this one.
https://gem.wiki/Yima_Gezha_power_station	China	Henan	Yima Gezha power station Unit 1	Yima Gezha power station	155	2010	2013	3	In 2013, due to the plant not meeting environmental standards, it went offline. The parent company Wuhan East Lake High Technology Group Co., Ltd is looking for buyers to take over. However, as of 2021 the plant is still offline, and is presumably retired.
https://gem.wiki/Yima_Gezha_power_station	China	Henan	Yima Gezha power station Unit 2	Yima Gezha power station	155	2009	2013	4	In 2013, due to the plant not meeting environmental standards, it went offline. The parent company Wuhan East Lake High Technology Group Co., Ltd is looking for buyers to take over. However, as of 2021 the plant is still offline, and is presumably retired.
https://gem.wiki/Henan_Zhongmai_Yong'an_power_station	China	Henan	Henan Zhongmai Yong'an power station Unit 1	Henan Zhongmai Yong'an power station	100	2005	2012	7	Based on satellite imagery, the plant appeared to stop operating in 2012

https://gem.wiki/Henan_Zhongmai_Yong'an_power_station	China	Henan	Henan Zhongmai Yong'an power station Unit 2	Henan Zhongmai Yong'an power station	100	2005	2012	7	Based on satellite imagery, the plant appeared to stop operating in 2012
https://gem.wiki/Henan_Xinwang_power_station	China	Henan	Henan Xinwang 1	Henan Xinwang power station	135	2006	2014	8	In 2014 the company, facing financial difficulty, retired the plant
https://gem.wiki/Henan_Xinwang_power_station	China	Henan	Henan Xinwang 2	Henan Xinwang power station	135	2007	2014	7	In 2014 the company, facing financial difficulty, retired the plant
https://gem.wiki/Henan_Shenhuo_power_station	China	Henan	Henan Shenhuo-2 power station Unit 2	Henan Shenhuo power station	135	2008	2017	9	No specific information on the reason for retirement provided
https://gem.wiki/Henan_Shenhuo_power_station	China	Henan	Henan Shenhuo-2 power station Unit 3	Henan Shenhuo power station	135	2008	2017	9	No specific information on the reason for retirement provided
https://gem.wiki/Hunan_Yongxing_power_station	China	Hunan	Hunan Yongxing power station Unit 1	Hunan Yongxing power station	60	2008	2014	6	No specific information on the reason for retirement provided
https://gem.wiki/Hunan_Yongxing_power_station	China	Hunan	Hunan Yongxing power station Unit 2	Hunan Yongxing power station	60	2009	2014	5	No specific information on the reason for retirement provided
https://gem.wiki/Nanjing_Huarun_Thermal_power_station	China	Jiangsu	Nanjing Huarun Thermal power station Unit 1	Nanjing Huarun Thermal power station	135	2004	2011	7	No specific information on the reason for retirement provided
https://gem.wiki/Nanjing_Huarun_Thermal_power_station	China	Jiangsu	Nanjing Huarun Thermal power station Unit 2	Nanjing Huarun Thermal power station	135	2004	2011	7	No specific information on the reason for retirement provided
https://gem.wiki/Huaneng_Suzhou_Cogen_power_station	China	Jiangsu	Huaneng Suzhou Cogen Power Station Unit 1	Huaneng Suzhou Cogen power station	60	2006	2014	8	No specific information on the reason for retirement provided
https://gem.wiki/Huaneng_Suzhou_Cogen_power_station	China	Jiangsu	Huaneng Suzhou Cogen Power Station Unit 2	Huaneng Suzhou Cogen power station	60	2006	2014	8	No specific information on the reason for retirement provided
https://gem.wiki/Nanjing-2_power_station	China	Jiangsu	Nanjing-2 Power Station Unit 3	Nanjing-2 power station	125	2008	2014	6	Part of the unit that first built in 1988. This unit follow the entire plant retirement in 2014.
https://gem.wiki/CPI_Fenyi_power_station	China	Jiangxi	Fenyi power station Unit 7	CPI Fenyi power station	100	2003	2011	8	No specific information on the reason for retirement provided

https://gem.wiki/Huaneng_Baishan_power_station	China	Jilin	Huaneng Baishan power station Unit 1	Huaneng Baishan power station	330	2011	2020	9	No specific information on the reason for retirement provided
https://gem.wiki/Huaneng_Baishan_power_station	China	Jilin	Huaneng Baishan power station Unit 2	Huaneng Baishan power station	330	2011	2020	9	No specific information on the reason for retirement provided
https://gem.wiki/Fushun_CPI_power_station	China	Liaoning	Fushun CPI power station Unit 2	Fushun CPI power station	200	2002	2011	9	No specific information on the reason for retirement provided
https://gem.wiki/Chentangzhuang_power_station	China	Tianjin	Chentangzhuang power station Unit 5	Chentangzhuang power station	135	2005	2010	5	No specific information on the reason for retirement provided
https://gem.wiki/Chentangzhuang_power_station	China	Tianjin	Chentangzhuang power station Unit 8	Chentangzhuang power station	300	2008	2015	7	No specific information on the reason for retirement provided
https://gem.wiki/Chentangzhuang_power_station	China	Tianjin	Chentangzhuang power station Unit 9	Chentangzhuang power station	300	2008	2015	7	No specific information on the reason for retirement provided
https://gem.wiki/Yulin_Jinlong_Cogen_power_station	China	Shaanxi	Yulin Jinlong Cogen power station unit 1	Yulin Jinlong Cogen power station	50	2008	2016	8	No specific information on the reason for retirement provided
https://gem.wiki/Yulin_Jinlong_Cogen_power_station	China	Shaanxi	Yulin Jinlong Cogen power station unit 2	Yulin Jinlong Cogen power station	50	2008	2016	8	No specific information on the reason for retirement provided
https://gem.wiki/Shandong_Chenming_Mill_power_station	China	Shandong	Shandong Chenming Mill power station Unit 3	Shandong Chenming Mill power station	155	2013	2018	5	No specific information on the reason for retirement provided
https://gem.wiki/Binzhou_Heating_Supply_Center_power_station	China	Shandong	Binzhou Heating Supply Center power station Unit 5	Binzhou Heating Supply Center power station	135	2013	2020	7	Plant operator Shandong Weiqiao Group was forced to close several plants and units including this one after the central government discovered the region had illegally built multiple plants.
https://gem.wiki/Binzhou_Heating_Supply_Center_power_station	China	Shandong	Binzhou Heating Supply Center power station Unit 6	Binzhou Heating Supply Center power station	135	2012	2020	8	Same as above

https://gem.wiki/Binzhou_Heating_Supply_Center_power_station	China	Shandong	Binzhou Heating Supply Center power station Unit 7	Binzhou Heating Supply Center power station	135	2012	2020	8	Same as above
https://gem.wiki/Binzhou_Heating_Supply_Center_power_station	China	Shandong	Binzhou Heating Supply Center power station Unit 8	Binzhou Heating Supply Center power station	135	2012	2020	8	Same as above
https://gem.wiki/Weiqiao_Aluminum_power_station	China	Shandong	Weiqiao Aluminum power station Unit 6-1	Weiqiao Aluminum power station	330	2012	2020	8	Same as above
https://gem.wiki/Weiqiao_Aluminum_power_station	China	Shandong	Weiqiao Aluminum power station Unit 6-2	Weiqiao Aluminum power station	330	2012	2020	8	Same as above
https://gem.wiki/Weiqiao_Aluminum_power_station	China	Shandong	Weiqiao Aluminum power station Unit 6-3	Weiqiao Aluminum power station	330	2012	2020	8	Same as above
https://gem.wiki/Weiqiao_Aluminum_power_station	China	Shandong	Weiqiao Aluminum power station Unit 6-4	Weiqiao Aluminum power station	330	2012	2020	8	Same as above
https://gem.wiki/Minjiang_power_station	China	Sichuan	Minjiang power station Unit 1	Minjiang power station	135	2006	2012	6	No specific information on the reason for retirement provided
https://gem.wiki/Minjiang_power_station	China	Sichuan	Minjiang power station Unit 2	Minjiang power station	135	2006	2012	6	No specific information on the reason for retirement provided
https://gem.wiki/Tashdian_power_station	China	Xinjiang	Tashdian power station Unit 8	Tashdian power station	125	2011	2019	8	No specific information on the reason for retirement provided

https://gem.wiki/Tashdian_power_station	China	Xinjiang	Tashdian power station Unit 9	Tashdian power station	125	2012	2019	7	No specific information on the reason for retirement provided
https://gem.wiki/Tianfu_East_power_station	China	Xinjiang	Tianfu East power station new unit 1	Tianfu East power station	135	2011	2018	7	No specific information on the reason for retirement provided
https://gem.wiki/Tianfu_East_power_station	China	Xinjiang	Tianfu East power station new unit 2	Tianfu East power station	135	2011	2018	7	No specific information on the reason for retirement provided
https://gem.wiki/Tianfu_East_power_station	China	Xinjiang	Tianfu East power station new unit 3	Tianfu East power station	135	2012	2018	6	No specific information on the reason for retirement provided
https://gem.wiki/Tianfu_West_power_station	China	Xinjiang	Tianfu West power station new Unit 1	Tianfu West power station	135	2013	2018	5	No specific information on the reason for retirement provided
https://gem.wiki/Tianfu_West_power_station	China	Xinjiang	Tianfu West power station new Unit 2	Tianfu West power station	135	2013	2018	5	No specific information on the reason for retirement provided
https://gem.wiki/Tianfu_West_power_station	China	Xinjiang	Tianfu West power station new Unit 3	Tianfu West power station	135	2013	2018	5	No specific information on the reason for retirement provided
https://gem.wiki/Urumuqi_Huanpeng_power_station	China	Xinjiang	Urumuqi Huanpeng power station Unit 3	Urumuqi Huanpeng power station	30	2009	2018	9	No specific information on the reason for retirement provided
https://gem.wiki/Urumuqi_Huanpeng_power_station	China	Xinjiang	Urumuqi Huanpeng power station Unit 4	Urumuqi Huanpeng power station	30	2009	2018	9	No specific information on the reason for retirement provided
https://gem.wiki/Albbruck_Mill_power_station	Germany	Baden-Wuerttemberg	Albbruck Mill New power station Unit 1	Albbruck Mill power station	31	2009	2012	3	No specific information on the reason for retirement provided
https://gem.wiki/Nakoso_power_station	Japan	Tōhoku	Nakoso power station Unit 10	Nakoso power station	250	2013	2020	7	This plant appears to have been retired to enable replacement with a larger and high-efficiency one
https://gem.wiki/Khabarovsk-3_power_station a	Russia	Khabarovsk	Khabarovsk-3 power station Unit 4	Khabarovsk-3 power station	180	2006	2012	6	No specific information on the reason for retirement provided
https://gem.wiki/Sandow_power_station	United States	Texas	Sandow Station Unit 5	Sandow power station	661.5	2009	2018	9	Units 4-5 were retired in January 2018 due to economic factors including low natural gas prices and growth in renewable energy

Analysis

Table S4. Hyperparameter tuning

Groups	Hyperparameter tuning*
China	Best Parameters: {'n_estimators': 70, 'min_samples_split': 6, 'min_samples_leaf': 1, 'max_features': 'auto', 'max_depth': 100, 'bootstrap': True}
USA	Best Parameters: {'n_estimators': 90, 'min_samples_split': 6, 'min_samples_leaf': 3, 'max_features': 'auto', 'max_depth': 90, 'bootstrap': True}
Rest of the World	Best Parameters: {'n_estimators': 75, 'min_samples_split': 6, 'min_samples_leaf': 3, 'max_features': 'auto', 'max_depth': 40, 'bootstrap': True}

*Note: Best parameters from Hyperparameter tuning will always change every time we run the script code. The result appears in the table is based on the specific time when the authors run the script code.

Table S5. Accuracy results

	Model with three groups			Single model		
	China	USA	RoW	China	USA	RoW
**Average retirement age**	22	45	33	26	44	39
**Max age**	44	60	56	36	60	60
**Min age**	13	37	13	16	37	11
**Accuracy = 100 - MAPE (Mean Absolute Percentage Error)**	82.13%	78.79%	78.53%	81.30%		
**Mean Absolute Error (MAE)**	3.71	7.87	6.41	5.6		
**Mean Square Error (MSE)**	32.26	132.07	106.2	68.4		
**Root Mean Squared Error (RMSE)**	5.68	11.49	10.31	8.27		
**Notes**	SHAP value results show CO2 emissions is the most influential factor in the model, exerting a clear magnitude on early or later retirements.			Note that here the accuracy result is a single number since this is a single model. Then, we analyzed retirement ages based on the three groups (China, USA, Rest of the World) to give an equal comparison.<br><br>SHAP value results shows that GDP is the most influential factor in the model, However, the magnitude of GDP's effect on retirement is not clear.		

Note: The Mean Square Error (MSE) determines the average squared differences between the prediction and true values in the regression. Prediction models with a lower MSE perform better. However, because it measures quadratic differences, the MSE is difficult to interpret. Instead, the Mean Absolute Error (MAE) and Mean Absolute Percentage Error (MAPE), which calculate the average absolute gap between predictions and true value, are helpful to measure predictive accuracy (Foster *et al.*, 2021). Therefore, we use accuracy as the main parameter to determine the sufficiency of the model. Accuracy more than 50% is considered sufficient, as explained in the Table S3.3.

Table S6. Classification of accuracy

**Accuracy = 100 - MAPE**	**Accuracy of forecast**
Less than 49%	Inaccurate forecast
50% - 79%	Reasonable forecast
80% - 89%	Good forecast
More than 90%	Highly accurate forecast

Source: Kasemset, Sae-Haew and Sopadang (2014)

Table S7. Predicted future retirement ages (unit numbers)

Country	Average unit age as of 2021 (years)	Average unit age as of 2025 (years)	Retire ≤40 years (units)	Retire >40 years (units)	Total units
Argentina	38	0	0	1	1
Australia	34	39	6	53	59
Bangladesh	1	5	14	2	16
Bosnia and Herzegovina	40	32	3	7	10
Brazil	24	34	7	10	17
Bulgaria	43	53	0	31	31
Cambodia	2	6	2	10	12
Canada	37	44	0	22	22
Chile	16	20	9	14	23
China	12	17	3003	10	3013
Colombia	33	32	3	11	14
Croatia	21	25	1	0	1
Czech Republic	47	56	20	47	67
Denmark	27	31	3	0	3
Dominican Republic	20	24	2	3	5
Finland	39	45	1	7	8
France	24	30	0	12	12
Germany	35	38	41	60	101
Greece	27	41	7	0	7
Guatemala	8	12	0	13	13
Honduras	5	9	0	3	3
Hungary	55	0	0	7	7
India	15	19	534	346	880
Indonesia	7	12	74	181	255
Ireland	35	40	3	0	3
Israel	31	38	0	10	10
Italy	32	42	10	8	18
Japan	22	26	50	107	157
Kazakhstan	41	47	0	91	91
Kyrgyzstan	37	29	0	9	9
Laos	6	10	3	0	3
Madagascar	10	14	0	3	3
Malaysia	14	18	17	8	25
Mexico	29	33	0	15	15
Mongolia	23	25	0	12	12
Montenegro	39	0	0	1	1
Morocco	24	34	9	5	14
Myanmar*	12	16	0	3	3
Netherlands	10	14	5	0	5
New Zealand	39	0	0	2	2
North Macedonia	38	41	1	3	4
Pakistan	2	6	0	20	20
Panama	7	11	0	5	5
Peru	22	26	0	1	1
Philippines	9	13	0	60	60

Poland	40	40	5	150	155
Romania	39	42	10	11	21
Russia	44	48	34	272	306
Senegal	5	9	1	1	2
Serbia	46	56	1	16	17
Slovakia	55	0	0	6	6
South Africa	38	49	39	64	103
South Korea	15	20	63	20	83
Spain	37	39	0	11	11
Sri Lanka	8	12	3	0	3
Tajikistan	6	10	0	4	4
Thailand	19	23	4	16	20
Turkey	19	22	53	25	78
Ukraine	54	63	0	93	93
United Arab Emirates*	0	4	4	0	4
United Kingdom	48	42	0	12	12
United States	43	47	26	453	479
Uzbekistan	40	60	7	5	12
Vietnam	9	13	54	25	79
Zambia	5	9	0	3	3
Zimbabwe	24	24	4	5	9

* All units are under construction

Table S8. Predicted future retirement age (capacity)

Country	Average units age as of 2021 (years)	Average units age as of 2025 (years)	Retire ≤40 years (MW)	Retire >40 years (MW)	Total capacity
Argentina	38	0	0	375	375
Australia	34	39	4,160	20,517	24,677
Bangladesh	1	5	8,329	250	8,579
Bosnia and Herzegovina	40	32	830	1,243	2,073
Brazil	24	34	2,503	674	3177
Bulgaria	43	53	0	4,829	4,829
Cambodia	2	6	700	1,020	1,720
Canada	37	44	0	5,679	5679.8
Chile	16	20	2,727	2,214	4,941
China	12	17	1,092,452	1,050	1,093,502
Colombia	33	32	601	1,032	1,633
Croatia	21	25	210	0	210
Czech Republic	47	56	4,630	2,775	7,405
Denmark	27	31	1,180	0	1,180
Dominican Republic	20	24	752	312	1,064
Finland	39	45	39	1,429	1,468
France	24	30	0	3,107	3,107
Germany	35	38	15,481	22,874	38,355
Greece	27	41	2,585	0	2,585
Guatemala	8	12	0	1,010	1,010
Honduras	5	9	0	105	105
Hungary	55	0	0	944	944
India	15	19	229,435	31,965	261400
Indonesia	7	12	36,231	14,594	50,825
Ireland	35	40	915	0	915
Israel	31	38	0	4,900	4,900
Italy	32	42	5,190	1,766	6,956
Japan	22	26	24,678	30,905	55,583
Kazakhstan	41	47	0	11,949	11,949
Kyrgyzstan	37	29	0	910	910
Laos	6	10	1,878	0	1,878
Madagascar	10	14	0	120	120
Malaysia	14	18	12,200	1,080	13,280
Mexico	29	33	0	5,378	5,378
Mongolia	23	25	0	1,010	1,010
Montenegro	39	0	0	225	225
Morocco	24	34	3,792	465	4,257
Myanmar*	12	16	0	160	160
Netherlands	10	14	4,152	0	4,152
New Zealand	39	0	0	500	500
North Macedonia	38	41	225	575	800

Pakistan	2	6	0	8,268	8,268
Panama	7	11	0	426	426
Peru	22	26	0	135	135
Philippines	9	13	0	12,178	12,178
Poland	40	40	4,643	25,636	30,279
Romania	39	42	1,800	2,875	4,675
Russia	44	48	10,068	31,361	41,429
Senegal	5	9	125	30	155
Serbia	46	56	670	3,735	4,405
Slovakia	55	0	0	561	561
South Africa	38	49	27,343	18,465	45,809
South Korea	15	20	40,602	1,692	42,294
Spain	37	39	0	3,089	3,089
Sri Lanka	8	12	900	0	900
Tajikistan	6	10	0	400	400
Thailand	19	23	1,762	4,226	5,988
Turkey	19	22	16,160	4,077	20,238
Ukraine	54	63	0	19,525	19,525
United Arab Emirates	0	4	2,400	0	2,400
United Kingdom	48	42	0	6,328	6,328
United States	43	47	12,381	214,596	226,977
Uzbekistan	40	60	2,100	393	2,493
Vietnam	9	13	26,132	2,225	28,357
Zambia	5	9	0	330	330
Zimbabwe	24	24	1,110	530	1,640

* All units are under construction

Table S9. Remaining lifetime CO_2 emissions

Country	Remaining lifetime CO2 after 2025 (reference scenario)	Remaining lifetime CO2 after 2025 (model prediction)	Discrepancy	Retired units in 2025 (reference scenario)	Retired units in 2025 (model prediction)	Total MW retired in 2025 (reference scenario)	Total MW retired in 2025 (model prediction)
Argentina	0	0	0%	0	1	-	375
Australia	1451.6	578.3	-60%	0	11	-	2,956
Bangladesh	1168.3	1153.8	-1%	0	0	-	-
Bosnia and Herzegovina	53.3	39.9	-25%	0	2	-	600
Brazil	305.5	280.3	-8%	0	3	-	207
Bulgaria	140.8	158.2	12%	2	7	120	802
Cambodia	256.3	296.6	16%	0	0	-	-
Canada	74.1	157.4	112%	0	10	-	2,691
Chile	162.4	524.2	223%	0	6	-	1,172
China	114844.8	49300.9	-57%	20	53	3,070	9,645
Colombia	110.3	107.6	-2%	0	3	-	380
Croatia	16.5	9.9	-40%	0	0	-	-
Czech Republic	307.5	288.2	-6%	0	7	-	868
Denmark	8	31.8	298%	0	2	-	769
Dominican Republic	115.2	119.9	4%	0	1	-	128
Finland	27	32.3	20%	0	3	-	405
France	33	92.7	181%	0	8	-	2,737
Germany	1591.6	1538.6	-3%	3	39	655	10,746
Greece	10.4	132	1169%	0	6	-	1,925
Guatemala	133	163.2	23%	0	0	-	-
Honduras	18.6	24	29%	0	0	-	-
Hungary	5.2	0	-100%	0	3	-	420
India	27150.3	24228.4	-11%	0	56	-	8,664
Indonesia	6095.6	6296.7	3%	0	2	-	800
Ireland	4.8	1.6	-67%	0	0	-	-

Country	Remaining lifetime CO2 after 2025 (reference scenario)	Remaining lifetime CO2 after 2025 (model prediction)	Discrepancy	Retired units in 2025 (reference scenario)	Retired units in 2025 (model prediction)	Total MW retired in 2025 (reference scenario)	Total MW retired in 2025 (model prediction)
Israel	0	184.3	0%	0	10	-	4,900
Italy	31	237.9	667%	0	2	-	336
Japan	3813	3948.6	4%	0	9	-	3,288
Kazakhstan	268.3	400	49%	0	15	-	2,978
Kyrgyzstan	0	68.6	0%	0	9	-	910
Laos	236.6	221	-7%	0	0	-	-
Madagascar	15.6	22.2	42%	0	0	-	-
Malaysia	953.4	1262.5	32%	0	0	-	-
Mexico	216	253.8	18%	0	0	-	-
Mongolia	50.6	67.2	33%	0	3	-	256
Montenegro	0	0	0%	0	1	-	225
Morocco	397.2	331.1	-17%	0	0	-	-
Myanmar*	18.1	24.7	36%	0	0	-	-
Netherlands	65.5	402.7	515%	0	1	-	652
New Zealand	2.8	0	-100%	0	0	-	-
North Macedonia	9.8	1.4	-86%	0	1	-	125
Pakistan	1140.8	1196.1	5%	0	0	-	-
Panama	8.4	75.6	800%	0	3	-	120
Peru	0	14.7	0%	0	1	-	135
Philippines	1403.4	1830	30%	0	2	-	340
Poland	1217.4	1444.9	19%	14	35	2,412	5,749
Romania	30.4	2.1	-93%	0	1	-	330
Russia	1018.7	1213.8	19%	0	36	-	4,620
Senegal	26.8	28.2	5%	0	0	-	-
Serbia	27.8	20	-28%	0	7	-	1,649
Slovakia	1.9	0	-100%	0	2	-	220

Country	Remaining lifetime CO2 after 2025 (reference scenario)	Remaining lifetime CO2 after 2025 (model prediction)	Discrepancy	Retired units in 2025 (reference scenario)	Retired units in 2025 (model prediction)	Total MW retired in 2025 (reference scenario)	Total MW retired in 2025 (model prediction)
South Africa	3454.8	1262.4	-63%	0	5	-	667
South Korea	2744	3823.6	39%	0	2	-	1,120
Spain	4.7	38.5	719%	0	7	-	2,735
Sri Lanka	111.8	99.8	-11%	0	0	-	-
Tajikistan	55	70.2	28%	0	0	-	-
Thailand	354.5	525.6	48%	0	1	-	300
Turkey	1794.4	1597	-11%	0	6	-	1,430
Ukraine	223.4	45.1	-80%	0	9	-	1,730
United Arab Emirates	314.6	286	-9%	0	0	-	-
United Kingdom	10.4	3.4	-67%	1	8	701	4,156
United States	5582.8	4162.8	-25%	0	109	-	47,522
Uzbekistan	63.1	51.7	-18%	0	2	-	600
Vietnam	3544.9	3553	0%	0	3	-	330
Zambia	46.4	50.9	10%	0	0	-	-
Zimbabwe	112.3	116.8	4%	0	3	-	360

Note: Reference scenario assumes a retirement age of 40 years. Model prediction is based on our machine learning prediction.

Table S10. Coal lock-in analysis

Country	Coal lock-in index	Total units in 2025	Total capacity in 2025
Mongolia	6.47	9	0
South Africa	5.22	98	21721
Kazakhstan	5.15	76	8579
India	5.07	824	1473
Indonesia	5.02	253	2970
Poland	5.02	120	4027
Philippines	4.60	58	1720
Bosnia and Herzegovina	4.59	8	2989.2
Cambodia	4.45	12	3769
Vietnam	4.06	76	1083857
Zimbabwe	4.03	6	1253.5
Czech Republic	3.79	60	210
Australia	3.75	48	6537.6
China	3.70	2960	411
Malaysia	3.61	25	936
Madagascar	3.60	3	1063.2
South Korea	3.58	81	370
Bulgaria	3.54	24	27609.4
Laos	3.51	3	660
Morocco	3.48	14	1010.3
Turkey	3.30	72	105
Japan	3.06	148	524
Greece	2.92	1	252736.7
Germany	2.75	62	50025.6
Serbia	2.72	10	915
Sri Lanka	2.71	3	0
United States	2.71	370	6620
Chile	2.65	17	52295.98
Netherlands	2.54	4	8971
Uzbekistan	2.49	10	0
Israel	2.43	0	1878
Thailand	2.43	19	120
Dominican Republic	2.42	4	13280
Russia	2.34	270	5378
Guatemala	2.33	13	754
Pakistan	2.23	20	0
North Macedonia	2.03	3	4257
Zambia	1.93	3	160
Ukraine	1.92	84	3500
Kyrgyzstan	1.75	0	500
Myanmar*	1.70	3	675
Colombia	1.63	11	8268
Denmark	1.50	1	306

Country	Coal lock-in index	Total units in 2025	Total capacity in 2025
Spain	1.47	4	0
Panama	1.41	2	11838
Italy	1.35	16	24530.6
Canada	1.34	12	4345
Croatia	1.28	1	36809.1
Mexico	1.20	15	155
Romania	1.16	20	2756
Brazil	1.10	14	341
Finland	1.04	5	45141.8
Bangladesh	1.03	16	41174
United Kingdom	0.68	4	355
Honduras	0.67	3	900
Tajikistan	0.66	4	400
France	0.66	4	5688
Senegal	0.64	2	18808.16
Ireland	0.62	3	17795
United Arab Emirates	0.61	4	2400
Peru	0.55	0	2172
Argentina	0.00	0	179456.06
Hungary	0.00	4	1893
Montenegro	0.00	0	28027
New Zealand	0.00	2	330
Slovakia	0.00	4	1280
