# Supplementary material for: Forecasting coal power plant retirement ages and lock-in with random forest regression
Source: Patterns (N Y). 2023 Jun 21;4(7):100776. doi: 10.1016/j.patter.2023.100776 (PMC10382988; doi:10.1016/j.patter.2023.100776)
Supplement: Document S2. Article plus supplemental information [file mmc2.pdf]

Article

Patterns

Forecasting coal power plant retirement ages and lock-in with random forest regression

Highlights

- Historical evidence could help identify factors influencing the retirement of CFPPs
- We identified the most important factors influencing retirement ages
- We forecast some countries to face considerable hurdles to retiring plants

Authors

Achmed Edianto, Gregory Trencher, Niccolò Manych, Kazuyo Matsubae

Correspondence

achmed.shahram.edianto.p1@alumni.tohoku.ac.jp (A.E.),
trencher.gregory.2s@kyoto-u.ac.jp (G.T.),
manych@mcc-berlin.net (N.M.)

In brief

We aim to determine the factors that influenced historical coal-fired power plant (CFPP) retirements and predict future retirement ages of existing and under-construction units. We also aim to forecast carbon emissions from CFPPs based on predicted retirement ages and then quantify the degree to which particular countries are locked in to coal power.

Edianto et al., 2023, Patterns *4*, 100776
July 14, 2023 © 2023 The Author(s).
https://doi.org/10.1016/j.patter.2023.100776

CellPress

Patterns

CellPress
OPEN ACCESS

Article

Forecasting coal power plant retirement ages and lock-in with random forest regression

Achmed Edianto,[1,5,*] Gregory Trencher,[2,*] Niccolò Manych,[3,4,*] and Kazuyo Matsubae[1]
[1]Graduate School of Environmental Studies, Tohoku University, Miyagi, Japan
[2]Graduate School of Global Environmental Studies, Kyoto University, Kyoto, Japan
[3]Mercator Research Institute on Global Commons and Climate Change, Berlin, Germany
[4]Department Economics of Climate Change, Technische Universität Berlin, Berlin, Germany
[5]Lead contact
*Correspondence: achmed.shahram.edianto.p1@alumni.tohoku.ac.jp (A.E.), trencher.gregory.2s@kyoto-u.ac.jp (G.T.), manych@mcc-berlin.net (N.M.)
https://doi.org/10.1016/j.patter.2023.100776

THE BIGGER PICTURE Coal power plants are the single largest contributor to global heating. With new constructions slowing globally, there is an urgent need to accelerate the retirement of existing plants. With numerous barriers hampering this, retirement speeds differ considerably across countries. It is thus unclear at what age existing plants will retire and where global hotspots of early or late retirements may be located. Our machine-learning model identifies plant-level CO_2 emissions and penetration of renewables in the electricity mix as the most important factors influencing retirement ages. Daring a look into the future brings good news: globally, most plants will likely retire earlier than the historical average of 40 years, generating large emissions reduction benefits. However, we forecast some countries will face considerable difficulties in retiring plants due to high lock-in, influenced by plant ages and dependence on coal. These countries merit targeted support by the international community.

1 2 3 4 5 **Development/Pre-production:** Data science output has been rolled out/validated across multiple domains/problems

SUMMARY

Averting dangerous climate change requires expediting the retirement of coal-fired power plants (CFPPs). Given multiple barriers hampering this, here we forecast the future retirement ages of the world's CFPPs. We use supervised machine learning to first learn from the past, determining the factors that influenced historical retirements. We then apply our model to a dataset of 6,541 operating or under-construction units in 66 countries. Based on results, we also forecast associated carbon emissions and the degree to which countries are locked in to coal power. Contrasting with the historical average of roughly 40 years over 2010–2021, our model forecasts earlier retirement for 63% of current CFPP units. This results in 38% less emissions than if assuming historical retirement trends. However, the lock-in index forecasts considerable difficulties to retire CFPPs early in countries with high dependence on coal power, a large capacity or number of units, and young plant ages.

INTRODUCTION

Coal power continues to exacerbate climate change and air pollution.[1] Bucking global efforts to phase-out or expedite retirements of coal-fired power plants (CFPPs), numerous countries have recently built new plants.[2,3] In addition to some 6,500 units totalling 2,000 GW of capacity that exist globally, another 450 GW of new construction risks to expand this fleet.[4] Once built, CFPPs typically operate for between 40 and 50 years.[5–7] If all existing and new plants achieved a similar lifetime, 278 Gt of CO_2 ($Gt\text{-}CO_2$) emissions would result (calculated by authors based on Global Energy Monitor (2022)). This would consume more than half of the remaining carbon budget (500 $Gt\text{-}CO_2$) permitted in scenarios that limit post-industrial warming to 1.5°C.[8] In parallel, air pollution and damage to human health would be prolonged for decades.[9]

This is an open access article under the CC BY license (http://creativecommons.org/licenses/by/4.0/).

New CFPP developments have recently slowed.[10] In 2021, newly installed capacity declined to less than half of 2015 levels, when the biggest installation occurred (see Figure S1; Table S1). Yet there remains an urgent and unsolved challenge of how to tackle the world's fleet of existing plants. This requires government interventions to accelerate the retirement of existing CFPPs across wealthy and developing economies[8,11] and knowledge of conditions that affect retirement ages.

Many obstacles are expected to impede efforts to expedite the retirement of CFPPs.[12–14] These can arise from resistance by industry, for whom early plant retirements can trigger financial losses,[15,16] but also from the political difficulty of provoking economic and societal disturbances across the power-generation and coal-extraction sector.[17] In addition, many countries lack the technological capacity[18,19] or resources to immediately shift to alternative energy technologies. Strategies are thus needed to confront these financial, political, and social factors that "lock in" coal power by hampering early plant retirement and thereby prolonging lifetime carbon emissions.[7,20]

Several studies have attempted to forecast retirement ages for the global CFPP fleet. But research is limited to top-down approaches, where retirement volumes or schedules are prescribed in accord with climate mitigation scenarios.[21–24] Moreover, the usefulness of top-down estimates is reduced by the tendency of governments and market actors to ignore carbon budgets when deciding retirement schedules. Meanwhile, existing studies employing a bottom-up approach are limited to those modeling retirement ages based on plant-by-plant conditions.[25] Conversely, although recent scholarship adds to our understanding of the factors that influenced historical CFPP lifetimes,[26] it is still unclear how these could affect retirement ages in the future. Consequently, we have a limited understanding of what operational lifetimes to anticipate for currently operating CFPPs, what emissions will result, and what countries will experience lock-in to coal power.

We therefore aim to determine the factors that influenced historical CFPP retirements and forecast future retirement ages based on these results. We also aim to estimate carbon emissions from CFPPs based on forecasted retirement ages and to quantify the degree to which countries are locked in to coal power. To achieve this, we first extracted from literature descriptions of factors expected to influence retirement ages. We then collected county-specific data for these from publicly available sources (World Bank [2018],[27] BP [2021],[28] EMBER [2021],[29] etc.). Next, we used supervised machine learning to measure the influence of these on retirement ages, examining 1,697 units retired between 2010 and 2020 in 34 countries. We subsequently apply the model to a dataset of 6,541 units still operating or under construction in 66 countries to forecast future retirement ages and resulting emissions. Finally, we develop a coal lock-in index based on forecasted retirement ages, the share of coal in the electricity mix, and total capacity.

This study makes three important contributions. First, by examining historical evidence and country-specific conditions, we propose a new approach to estimate future CFPP lifetimes and ensuing emissions. Second, by identifying the factors that influenced historical retirements, we generate hints on conditions that could accelerate future retirements. Third, our novel lock-in index identifies countries meriting attention by policymakers and investors because of their forecasted challenges in phasing out coal.

The results section presents results of the machine-learning forecasting, estimated emissions, and the coal lock-in index and describes our data sources, sample constructions, and calculation procedures. The following section is the discussion, and the final section details the experimental procedures.

RESULTS

Historical drivers of early retirement

Our systematic review of academic literature led to the identification of 1 plant-level and 13 country-level factors of which data availability permitted their inclusion in our analysis of the historical drivers of CFPP retirement. Country-level factors describe the socio-economic, environmental, and governance conditions in a specific year, whereas plant-level factors reflect the characteristics of a CFPP unit (see Table 1 for all factors examined along with data sources).

Results of the Random Forest Regression used in the historical analysis are shown in Figure 1. Values appear in descending order, with the most important factors appearing as bars at the top of figures. To identify each factor's contribution to a unit's retirement, we used Shapley Additive Explanation (SHAP) values. A positive SHAP value indicates that a factor extends a unit's lifetime operation, whereas a negative value shortens it. The horizontal location on the bottom three figures indicates whether an SHAP value is associated with a higher or lower retirement age.

For China and the United States, a correlation implies that the retirement in a certain year is influenced by the value of a factor in that respective year. Results for the rest of the world (ROW), although based on country-specific data, provide only an average collective result for all 64 countries in the group. For example, if GDP data in country X and Y exerted a strong effect on early retirement, this would show up with a high SHAP value only if other countries in the ROW group experienced a similar situation. In contrast, results shown for China or the United States are based only on data from each of these countries.

For most countries with the exception of China, results show that a unit's annual CO_2 emissions (F1)—a plant-level factor based on a unit's capacity, heat rate, and type of coal combusted (see Table 1)—exert the highest impact on retirement ages, which in this case translates into a shorter lifetime. We thus find that countries have tended to prioritize the retirement of the most emissions-intensive units. Corroborating the findings of other studies,[30,31] this result suggests that the high fuel requirements of old and inefficient plants, which often require installation of expensive anti-pollution technologies, have provided an economic rationale for prioritized retirement. In both the United States and ROW, high annual emissions exert a strong negative impact on retirement, which in this case translates into a shorter lifetime. This can be seen in the graph where red and blue show the high or low value of the factor, whereas the x axis shows the negative (early retirement) or positive impact (late retirement) on the model. In contrast, China's results do not exhibit a clear correlation between annual CO_2 emissions and retirement age. A key factor influencing this unclear correlation is the low historical average retirement age, around 20 years,

Table 1. Country-level and plant-level factors that influence CFPP development and retirement

Factors	Data availability	Indicator unit	Source	Time-series data availability	Literature	Assumed influence	Notes
Included in analysis							
F1: Plant CO_2 emissions	yes	million tons CO_2/year	Global Energy Monitor (2022)[4]	yes	Trencher et al.,[30] Hughes et al.[31]	Plants with higher emissions are typically older plants with less-efficient technologies. Due to higher fuel requirements and a vulnerability to environmental or climate regulations, retirement schedules typically prioritize the most polluting plants.	Annual CO_2 emissions indicate absolute volumes, based on a unit's capacity, heat rate, and type of coal combusted. The heat rate is calculated from the coal combustion technology used (i.e., subcritical, supercritical, ultra-supercritical), as well as carbon capture and storage (CCS).
F2: Carbon price	yes	yes/no	World Bank (2018)[27]	no	Trencher et al.,[30] Mo et al.,[32] Pahle et al.,[33] Ross,[34] Mo et al.[35]	Carbon price policies increase the cost of coal-fired power generation, reducing competitiveness against other energy sources.	Countries indicated as possessing a carbon pricing policy during 2010–2020 receive a score of "1" and "0" if not.
F3: Coal price	yes	US$/ton	BP (2021)[28]	yes	Trencher et al.,[30] Mo et al.,[32] Pahle et al.[33]	A high average price of coal increases electricity generation costs, reducing competitiveness. Conversely, low prices increase competitiveness.	Average price for 2010–2020 used for Northwest Europe, US Central Appalachian, and Japan steam CIF.

(*Continued on next page*)

CellPress
OPEN ACCESS

Table 1. *Continued*

Factors	Data availability	Indicator unit	Source	Time-series data availability	Literature	Assumed influence	Notes
F4: Electricity access rate	yes	% of population with access to electricity	World Bank (2021)[36]	yes	Manych and Jakob[16]	Low electrification rates create a need to expand the electricity supply. Many developing countries choose CFPPs because they are an established and reliable technology, and they can supply large and stable baseload power.	Training set: Since 2020 data are unavailable and only one country in 2019 has not reached 100% (India); we assume that in 2020 all countries in this sample will reach 100% because India already reached 98% in 2019.<br><br>Model application set: Since 2020 data are not available, we extrapolate earlier data to forecast the 2020 share for a country that has not reached 100% access.
F5: Reliability of supply	yes	1–8	World Bank (2021)[37]	yes	Manych and Jakob[16]	Low reliability of electricity supply would drive the effort to provide stable electricity. In such conditions, CFPPs may be attractive because they are an established and reliable technology, and they can supply large and stable baseload power.	Since 2020 data are not available, we use 2019 because of the insignificant change between 2018 and 2019.
F6: GDP per capita	yes	US$/capita	World Bank (2021)[36]	yes	Scholvin,[38] Hao Tan et al.[39]	Poor economic conditions in a country create a need for cheap energy sources. The cheap fuel costs for CFPPs may encourage new constructions or delay retirements.	

(*Continued on next page*)

Table 1. *Continued*

Factors	Data availability	Indicator unit	Source	Time-series data availability	Literature	Assumed influence	Notes
F7: Renewable support policy	yes	1–100	World Bank (2021)[40]	yes	Gallagher et al.[41]	Policies that support renewable energy would drive deployment, decreasing costs over time, pushing coal out of power mixes because of low generation costs.	Based on the pillar "renewable energy" in Regulatory Indicators for Sustainable Energy (RISE).<br><br>Since 2020 data are not available, we extrapolate historical data.
F8: Climate policy effectiveness	yes	1–100	Wendling et al., (2020)[42]	yes	Scholvin,[38] Wang et al.[43]	Strict environmental policies may prevent the construction of new plants or induce the retirement of existing plants. They may also mandate expensive antipollution technologies, encouraging CFPPs to retire.	We use the performance score for "Climate Change" extracted from the environmental performance index (EPI) by Yale University to estimate the effectiveness of climate policies. Countries with strict or effective policy tend to have a lower carbon intensity score.
F9: Electricity demand growth	yes	% of electricity demand growth	EMBER (2021)[29]	yes	Manych and Jakob,[16] Dorband et al.[44]	A high growth of electricity demand creates a need to increase power production and drive the acceleration of affordable technologies with a large generation capacity. Under such conditions, CFPP can be competitive.	Model application set:<br>For countries without 2020 data, we extrapolate data from 2015 to 2019.<br><br>For Myanmar, the latest data are 2014, so we use the average growth of 2010–2014.

(*Continued on next page*)

Table 1. *Continued*

Factors	Data availability	Indicator unit	Source	Time-series data availability	Literature	Assumed influence	Notes
F10: Political stability	yes	−2.5 (weak) to 2.5 (strong)	World Bank (2021)[45]	yes	Scholvin,[38] Hao et al.[39]	Unstable political or socio-economic conditions create high uncertainty, especially for new technologies such as renewable energy that need supportive policies. In such conditions, CFPPs may be attractive.	Since 2020 data are not available, we extrapolate data from 2010 to 2019.
F11: Coal rent	yes	% of GDP	World Bank (2021)[36]	yes	Scholvin,[38] Blondeel et al.[46]	The availability of coal in a country increases the attractiveness of coal as an electricity source and would discourage the retirement of CFPPs.	We use coal rents to show the contribution of coal mining activity to a country's economy. High rents indicate active coal production with high revenue for a country. Coal reserves alone could not reflect this, because having large reserves without significant mining activity will not likely affect national coal policy. Since 2020 data are not available, we extrapolate data from 2015 to 2019.
F12: Natural gas price	yes	US$/mmBtu	BP (2021)[28]	yes	Ross,[34] Scholvin,[38] Fell and Kaffine,[47] Gray and Bernell[48]	A low average price of natural gas decreases the production cost of gas generation, reducing the competitiveness of CFPPs and encouraging it to retire.	We use the US Henry Hub natural gas price uniformly across all countries.

(*Continued on next page*)

Table 1. ***Continued***

Factors	Data availability	Indicator unit	Source	Time-series data availability	Literature	Assumed influence	Notes
F13: Renewable electricity output	yes	% of total power generation	EMBER (2021)[29] and IRENA (2021)[49]	yes	Dodd and Nelson,[50] Pahle et al.,[33] Fell and Kaffine[47]	A high penetration of renewable energy would decrease coal generation, reducing the utilization of CFPPs and encouraging retirement.	For 2010–2019 data, we use EMBER data because the latest data are from 2019 for most countries. We then use data from IRENA to cover 2020 data that are not available for some countries.
F14: Share of nuclear in electricity mix	yes	% of total power generation	EMBER (2021)[29]	yes	Trencher et al.,[30] Pahle et al.[33]	A high penetration of nuclear may decrease reliance on coal for baseload electricity and may also receive preference over coal because of its emission-free status.	
Excluded in analysis							
Plant age	yes	–	Global Energy Monitor	yes	Mo et al.,[32] Trencher et al.,[51] Dodd and Nelson[50]	–	–
Energy mix target	yes	–	NDC	no	Gallagher et al.,[41] Blondeel et al.,[46] Gray and Bernell[48]	–	–
Levelized cost of electricity (LCOE)	no	–	–	no	Trencher et al.,[30] Gallagher et al.,[41] Webb et al.,[52] Fell and Kaffine[53]	–	–
Government GHG emission reduction target	yes	–	NDC	no	Gallagher et al.,[41] Dorband et al.[44]	–	–
Coal support policy	no	–	–	no	Trencher et al.,[51] Pahle et al.[33]	–	–
Societal awareness	no	–	–	no	Trencher et al.[51]	–	–
State capacity	no	–	–	no	Brutschin et al.[26]	–	–

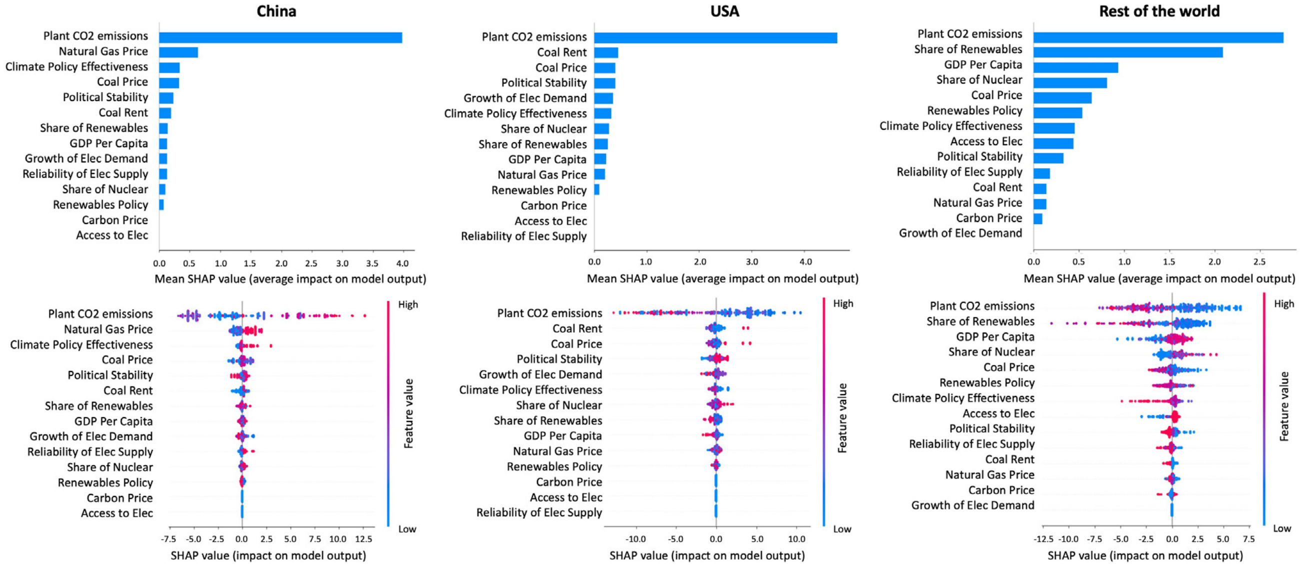

Figure 1. The impact of plant-level and country-level factors on forecasting a unit's retirement age in the historical analysis

Figures show corresponding SHAP values (top) and results for the SHAP correlation analysis (bottom). SHAP values indicate the mean value of the magnitude of each factor without explaining its positive or negative impact on the model. Feature values, indicated in red and blue (bottom), show the high or low value of the factor, while the x axis shows the negative (early retirement) or positive impact (late retirement) on the model. Feature value is different from the SHAP value and represents the "real value" of each factor. That is, while the SHAP value represents the mean value of the magnitude of that factor's influence on the model, the feature value shows the magnitude of that factor's impact on the model output, which in this case results in a higher or lower retirement age. For example, in the rest of the world (ROW), the feature value of plant CO_2 emissions is "high" (represented by red color), and the SHAP value magnitude is negative (represented by a negative value, left side of the chart). This situation can be translated as "high plant emissions will lead to an earlier retirement age." Explanations of each factor appear in Table 1. We divided the sample into three groups, China, the United States, and the ROW, to account for the large share of retired units by the first two countries, which make up 510 and 588 units, respectively (65% of the sample). Names on the y axis have been simplified for readability and differ slightly from those used in the script.

compared with other countries. Historically, Chinese coal plants usually have had a shorter lifespan than other countries, largely because of initiatives to close down smaller, dirtier power stations so as to better the air quality in the vicinity.[54]

Besides CO_2 emissions, although having less impact, some factors are still useful for the model's forecasting performance. In the case of China and the United States, other factors exert only a minor influence on plant retirement ages, including natural gas prices, carbon intensity, and coal rent. In China, for example, higher natural gas price could potentially extend the lifetime of CFPPs. However, the magnitude of these factors is close to zero, thus exerting almost no impact to the overall model.

The ROW group reveals an especially strong influence for a country's share of renewable electricity (F13) and GDP. This indicates that the growth of renewables has induced early retirements by gradually squeezing coal out of the electricity mix. We presume this tendency to be most pronounced in the advanced economies in the ROW group.

Conversely, country-level factors found to exert the weakest influence on retirement ages are carbon price (F2), access to electricity (F4), and the reliability of electricity supply (F5) for China and the United States and electricity demand growth (F9) for the ROW. The heterogeneous impact of factors in the three country groups occurred because of their country-level nature. For China and the United States, we compare the changing influence of factors on retirement ages over time. In the ROW, however, we compare factors between countries over time.

Forecasted retirement ages

The forecasted retirement ages for 66 countries, based on results of the historical analysis, appear in Figure 2. The complete list of each country's retirement probability appears in Tables S7 and S8. We set 40 years as the threshold retirement age, because this reflects the average over 2010–2020[4] and is widely used in the literature.[5,6] Forty years thus provides a rough indicator of what could be considered an "early" retirement from a historical perspective.

Our model forecasts that 63% of units will be retired at less than 40 years. Such units equate to around 1,600 GW or 68% of global capacity. A massive proportion of early retirement occurs in China, which owns 46% of global units (3,003 of 6,541 units). Interestingly, our model forecasts extensive early retirements in China despite its extremely young fleet (average unit age in 2021 is only 12 years compared with a global average age of 26 years).

On the other hand, there are 47 countries (70% of sampled countries) that share a high probability (>50%) that unit(s) will be retired later than 40 years. Illustrated by rapidly developing Asian economies, such as Indonesia, Mongolia, Pakistan, and the Philippines, these countries all share the feature of having built new CFPPs in recent years, propelled by the need to deliver affordable electricity to meet rapidly growing demand.[12] A further three countries meriting attention are the United States, Japan, and Australia, where more than 50% of CFPP capacity is expected to retire later than the global average. More specifically, the share of units forecast to operate beyond 40 years in

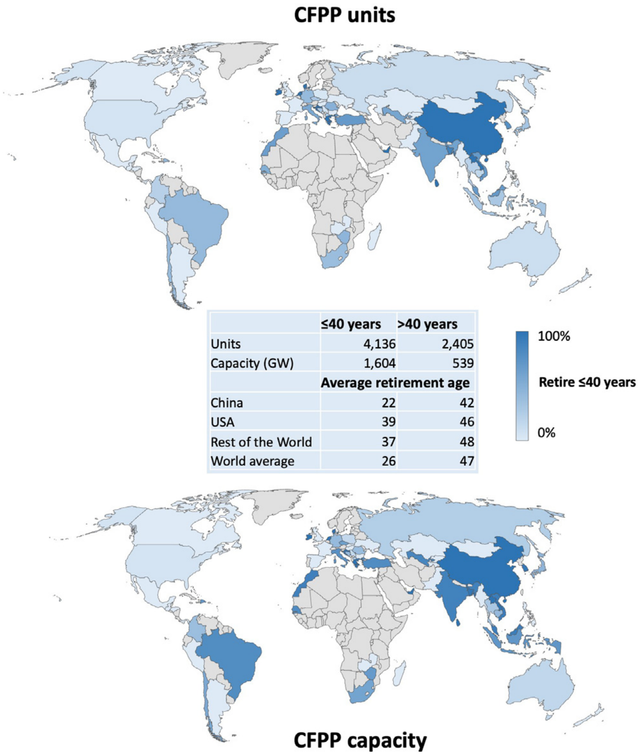

Figure 2. The forecasted retirement age of operating and under-construction CFPP units (n = 6,541) from 66 countries in terms of number of units (top) and capacity (MW) (bottom)

The map shows the cumulative units forecasted to retire before or after 40 years (the average global retirement age over 2010–2020). A country with a 100% value indicates that all CFPPs units will be retired before 40 years.

each of these countries is 95% (215 GW) of the 227 GW fleet in the United States, 56% (30 GW) of the 55 GW fleet in Japan, and 83% (20 GW) of the 25 GW fleet in Australia.

In this step of the analysis, we forecast the retirement ages for active units in each country and later calculate the year of retirement based on the year each unit begins operation. We anticipate that countries with a young fleet or with units still under construction will face hurdles to early retirements. For developing economies in Asia and atypical cases like Japan (Japan is an industrialized country with a young CFPP fleet (average age 22 years) that has seen multiple new additions in recent years.[51]), in the absence of policy interventions to prevent retirement ages mirroring historical trends, our model forecasts that many plants may operate beyond 2050, a year when many countries are aiming to reach carbon neutrality.

Forecasted emissions

We estimate in Figure 3 the total CO_2 emissions that would occur over the remaining lifetime for all CFPP units operating in 2025, the year by which we assume all plants under construction in our sample to come online. Although other factors such as changes in operational hours would affect future emissions, we focus on operational lifespans, because this is widely recognized as the greatest determinant of lifetime emissions.[32,50,51] This scenario also assumes the plant retirement ages forecasted by the machine-learning model. Resulting emissions are compared with a reference scenario. This uses either the planned retirement ages reported by plant operators (n = 804) or, for units without this information, an assumed lifetime of 40 years (n = 5,737), based on the average retirement age over 2010–2020.[4] The average retirement age forecasted by the model is 33 years, considerably shorter than in the reference scenario, which is 40 years.

The analysis indicates that plant retirements at the ages forecasted by our model would generate 114.5 Gt-CO_2, 38% less than the reference scenario. This reduction potential is considerable, being the equivalent of approximately 2 years' worth of annual global CO_2 emissions from all fossil fuels, which was 36.4 Gt in 2021.[55] This result suggests that future emissions from coal power may be significantly less than studies that assume plant lifetimes of around 40 years, based on historical trends.[7,24] Moreover, this finding demonstrates that expediting CFPP retirements can tremendously reduce global CO_2 emissions. However, this reduction is mainly contributed by China, which exhibits 57% less emissions than in the reference scenario. We also find that expedited retirements would lead to a considerable emissions reduction in the United States, being 25% less than the reference scenario. The ROW, meanwhile, exhibits a reduction potential of only 3%, because of younger plant ages in many countries.

Out of 66 countries, 27 will potentially see less future emissions than in the reference scenario (see Table S9). Conversely, the rest of the sample shows increased emissions. However, since these countries contribute only 13% of total emissions in the reference scenario, total emissions in the model forecasting do not increase.

Among the top emitters, China, the United States, India, and Australia are forecasted to see considerable carbon reductions relative to the reference scenario. These are largest for Australia and China (60% and 57%, respectively), whereas the United States and India may see 25% and 11% fewer emissions. China's potential for a massive reduction (65 Gt-CO_2) reflects the high probability for CFPPs to be retired below the age of 40 years. For the United States, the emissions reduction is based on our forecasting that 109 units will be retired in 2025, compared with zero units in the reference scenario. In Australia, although the model forecasts only 17% of CFPP capacity will be retired before 40 years, future emissions are 60% less than in the reference scenario. This result reflects the old age of Australia's fleet (34 years) and the forecasting that most units will be retired by 2025.

Conversely, other high emitters such as Indonesia, Japan, the Philippines, Poland, and South Korea are forecasted to see higher lifetime emissions relative to the reference scenario. South Korea, the Philippines, and Poland incur the most significant increase, with 39%, 30%, and 19%, respectively. Meanwhile, Indonesia and Japan see an increase of only 3% and 4%. In Indonesia and South Korea, although the retirement forecasting analysis shows that 71% and 96% of capacity will retire before 40 years, emissions in these countries increase because of our model forecasting average retirement ages that are later than the reference scenario. Concretely, the model forecasts an average retirement age of 35 years in Indonesia and 21 years for South Korea compared with 28 and 16 years, respectively, in the reference scenario.

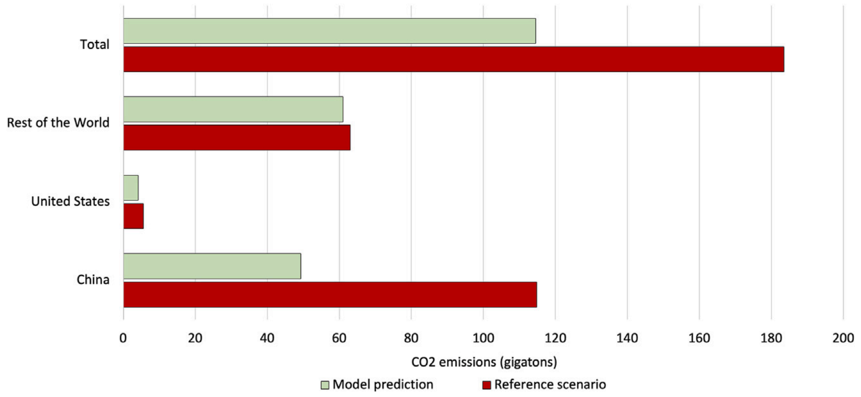

Figure 3. Remaining lifetime CO_2 emissions after 2025.

Coal lock-in index

Results for the coal lock-in index appear in Figure 4 and Table S10. Depicting the degree of difficulty each country is forecasted to face when attempting to retire its CFPP fleet, this index considers the CFPP fleet's age (based on the first year of commercial operation and the retirement age forecasted by the machine-learning analysis), the share of coal in the electricity mix, and the capacity of operating and under-construction CFPPs in the base year, 2025. Our coal lock-in index extends on the carbon lock-in equation proposed by Neofytou et al.,[56] integrating the forecasted retirement age into the model in place of a flat 40 years (see the detail explanation in experimental procedures).

Shown on the y axis, high numbers (e.g., 6) reflect a high degree of lock-in to coal power, whereas low numbers (e.g., 1) reflect a low degree of lock-in. To facilitate comparison, we organize individual country results in terms of total units (y axis) and capacity (size of circles). Four clusters of countries can be discerned from this analysis.

First are countries with a low lock-in index and a small volume of CFPP units, home to the vast majority of so-called coal phase-out countries. This group is characterized by countries such as France, Germany, New Zealand, Montenegro, Ireland, Spain, and the United Kingdom.[57] Countries in this cluster have already retired many coal plants in the past while adding few new ones, which decreases hurdles to retiring additional units. In the case of New Zealand, the lock-in index is zero, but the country still has two CFPP units, each sized 500 MW, and they are relatively old, commissioned in the 1980s. Here, however, the analysis shows that New Zealand is not likely to encounter any difficulty in eliminating coal from its power mix.

Second are countries with a small volume of CFPP units but a high degree of lock-in. This group includes Mongolia, Cambodia, and Bosnia, in addition to other developing economies still immersed in the "phase-in" stage; namely, Vietnam and the Philippines.[57] In the case of Mongolia, although the number of units (12) and CFPP capacity (1010 MW) is low compared with other countries, the lock-in score (6.47) is the highest of all countries. This result especially reflects two conditions: a high share of coal in the electricity mix (93% in 2025) and a tendency for plants to operate for more than 40 years. With a further two more units scheduled to come online, Mongolia's heavy reliance on coal power is expected to pose formidable barriers to expediting plant retirements.

Third are countries with a larger number of units but lower lock-in. Featuring industrialized giants such as Japan and the United States, in addition to Russia, coal lock-in scores in this group are below the median, between 2.34 and 3.06. Nonetheless, the significant volume of CFPP units in these countries is expected to hamper future efforts to expedite retirements. Consider the case of Japan, whose coal lock-in score is 3.06. This is due to a roughly 30% share of coal in the electricity mix and a forecasted average lifetime operation of CFPPs close to 42 years. However, Japan has a slightly higher lock-in than the United States and Russia. This reflects its larger volume of CFPP units and much younger fleet age, which is forecasted to average 26 years in 2025, the youngest among G7 countries.

Fourth is a cluster of five countries characterized by considerably more CFPP units and a high degree of lock-in: China, Indonesia, India, Poland, and South Africa. These countries are considered established coal users, some of which additionally export large quantities, which can result in increased reliance on coal from an economic perspective. In the case of China, although not featuring in the 10 most highly locked-in countries, we nonetheless anticipate considerable difficulty in completely phasing out coal power compared with other countries. This is due to the sheer volume of units and capacity. Indeed, although China's lock-in score is 3.7 and far below India, in 2025 this country will possess more than 1,000 GW, just over half (51%) of global capacity. India and Indonesia are other high lock-in countries with similar characteristics. India has the world's second largest CFPP fleet in terms of units and capacity, whereas Indonesia has the fifth highest number of units. Both also feature among the 10 countries with the highest coal lock-in score because of high shares of coal-fired electricity (74% in India and almost 60% in Indonesia). In addition, the high lock-in score for China, India, and Indonesia is also influenced by recently added or still-under-construction CFPPs. Indeed, these countries are home to the three largest CFPP pipelines globally, with 92, 31, and 15 GW under construction, respectively.

Of the four clusters identified, we argue that efforts to phase out coal power should focus on those countries with a high coal lock-in score and large unit numbers, namely, China, Indonesia, India, Poland, and South Africa, collectively representing more than two-thirds (68%) of global capacity in 2021. Retirement schedules in these countries will significantly influence global efforts to eliminate carbon emissions from electricity generation. As our lock-in result shows, barriers to this can arise not only from the share of coal in the power mix or from operational lifetimes, but also from the volume of units and capacity needing to be retired.

DISCUSSION

To mitigate climate change in line with temperature targets set under the Paris Agreement, halting the construction of CFPPs is essential but not enough; a timely retirement of existing plants

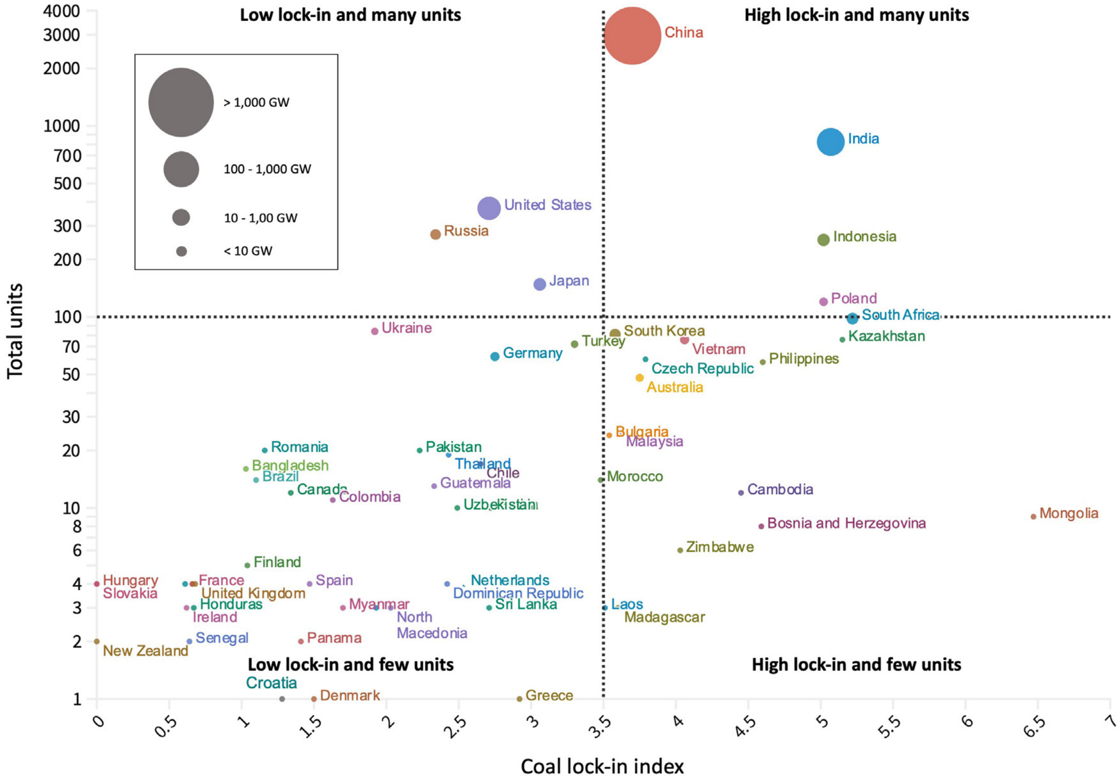

Figure 4. Results of coal lock-in index organized into total units (operating and under construction) and capacity (MW) among 66 countries

The size of circles represents the total capacity size (MW) in 2025 of each country, based on our model forecasting.

is also needed. This study focused on this latter challenge, using supervised machine-learning to identify factors that contributed to historical retirements over 2010–2020. We then applied the results to a dataset of 6,541 units in 66 countries to forecast future retirement ages, resulting emissions, and lock-in to coal power.

The historical analysis revealed that in all country groups, plant-level annual CO_2 emissions are the most important factor influencing retirement ages over 2010–2020. For countries other than China and the United States, we additionally found that the penetration of renewables in the electricity mix exerts a strong influence on early plant retirement. On the one hand, these results indicate that most countries are prioritizing the retirement of emissions-intensive units, which typically provides an economic rationale to do so. On the other hand, we find that renewable energy, which enjoys few operational costs, is gradually squeezing coal out of the power mix in many countries, inducing early retirements.

Although the average global retirement age over 2010–2021 has been around 40 years, our model suggests that many CFPPs will retire earlier in coming years, with 63% of units forecasted to retire earlier than the historical average. We also showed that earlier retirements have potential to generate a tremendous reduction in global CO_2 emissions, in this case, 38% less than a reference scenario assuming the 40-year historical average. The emissions-saving potential was highest in China and the United States. Conversely, the model also forecasts that plants will retire later than 40 years for many countries, especially Asian countries that have recently expanded or upgraded CFPP fleets. Without policy interventions to prevent this, numerous plants around the world are forecasted to operate beyond 2050, the year that many countries are aiming for carbon neutrality.

Shown by our analysis on coal lock-in, we forecast considerable difficulties in achieving early retirements in many countries, especially those characterized by high dependence on coal power, large capacity or unit numbers, and young fleets. This cluster includes China, Indonesia, India, Poland, and South Africa. Not only do these countries represent nearly 70% of worldwide coal power capacity in 2021, but China, India, and Indonesia are also home to the largest pipelines of CFPP developments.

These findings carry important policy implications. Many researchers and practitioners emphasize the need to phase out coal power from a global perspective or from the context of individual countries. However, our lock-in analysis reveals a need to focus attention on countries forecasted to experience considerable difficulty in implementing early retirements. These countries include China, Indonesia, India, Poland, and South Africa, all possessing high volumes of capacity and CFPP units, as well as strong lock-in. But other countries meriting special assistance in transitioning beyond coal are developing countries, such as Mongolia and Kazakhstan, also forecasted to suffer from coal lock-in despite smaller fleet sizes.

Our findings contribute to the growing literature taking interest in the speed at which coal phase-outs can occur. Although our results and approach are not directly comparable with other studies, there are several points of concurrence or differences between our study and others that merit brief mentioning. First, prioritizing the retirement of the most-polluting plants is the primary factor in our analysis

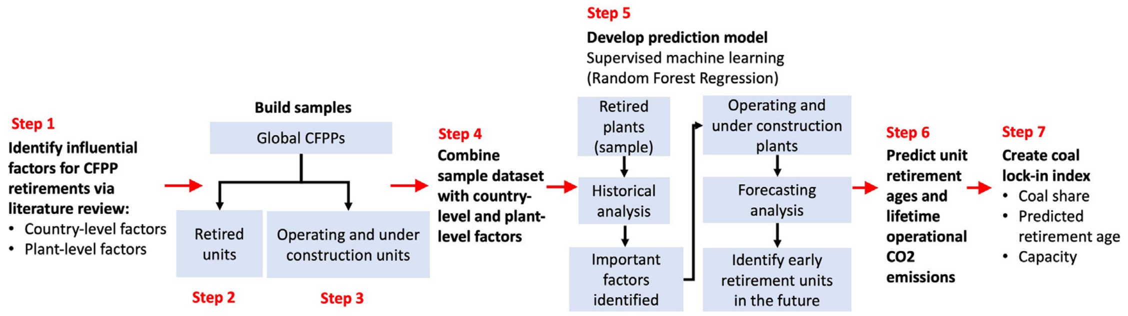

Figure 5. Summary of the study design.

and makes sense economically and for reducing emissions.[22] Second, the strictness of climate policies and government or industry ambitions to reduce dependance on coal will greatly determine retirement speeds.[26] Our model finds "Climate Policy Effectiveness" and "Renewables Policy" to be important, whereas other papers explicitly highlight the effect of carbon pricing on accelerating retirements.[25,35] Third, historical evidence shows that coal phase-outs are slow, require several decades, and typically occur faster in richer countries,[14,58] which is in line with our coal-index results. This once again points to the need for international collaboration to assist poorer countries with higher lock-in to expedite retirement schedules and replace coal with clean energy.

Several limitations in this study could inform future research. First, our analysis did not consider unit ages, because this is the output of the model. Since young plant ages are widely expected to hinder early retirement because of the need to recuperate sunken capital,[24] future studies could develop models capable of incorporating such plant-level factors. Data availability also impacted our application of other indicators. For example, for carbon pricing (F2), we considered the existence of such policies (yes/no) only because of the absence of more descriptive time-series data for the entire sample of countries. Second, questions also remain about the influence of certain variables identified from literature review, but for which data were not fully available (Table 1). These notably include plant age, the composition of power mix targets, levelized cost of electricity (LCOE), the ambition of government GHG emissions reduction targets, coal support policy, and societal awareness. Third, the importance of the influencing factors examined might change over the considered timescale, because many are affected by ever-changing dynamics in the energy market. Furthermore, we expect other future developments to influence plant retirement ages. For example, retrofitting plants with co-firing (ammonia, biomass) or carbon capture technologies would involve new investments that marginally reduce emissions, thus incentivizing longer operation. Finally, we also did not take into account coal phase-out pledges from governments, which in many countries will likely induce earlier retirements.[2] Including such factors could increase the robustness of forecasting results.

EXPERIMENTAL PROCEDURES

Resource availability

Lead contact

Further information and requests for resources should be directed to and will be fulfilled by the lead contact, Achmed Edianto (achmed.shahram.edianto.p1@alumni.tohoku.ac.jp).

Materials availability

This study did not generate new unique reagents.

Data and code availability

The publications comprising our sample, our coding procedure, and coding results are publicly available on the Zenodo platform.[59]

Our study design involves seven steps that we summarize in Figure 5 and elaborate below.

Systematic literature review

In the first step, we systematically reviewed academic literature to identify factors expected to drive or impede the retirement and development of CFPPs. We identified relevant studies from Scopus using a procedure explained in Notes S1.

The literature review led to the identification of 18 country-level and two plant-level factors (Table 1). Country-level factors describe the socio-economic, environmental, and governance conditions in a specific year, whereas plant-level factors reflect the characteristics of a CFPP unit (e.g., capacity, emissions, and technology). Due to data availability, we limit the factors used for the forecasting analysis to 13 of these.

Annual CO_2 emissions from each CFPP unit (F1) is the only plant-level factor included in the model. Inclusion of this factor assumes that plants with higher emissions are typically older plants with less-efficient technologies. Having higher fuel requirements and a vulnerability to environmental or climate regulations, retirement schedules typically prioritize the most polluting plants.

Besides this factor, plant age, determined by the commercial operation date (COD), often appeared in the literature based on views that old plants are more likely to retire than young plants. However, our forecasting model could not use this factor for two reasons. First, the training sample is biased compared with the overall sample. Specifically, only older plants can be retired at old ages, which is simply not feasible for younger ones. Second, the model's output is a unit's retirement age, which essentially reflects a plant's age at that point in time. This differs from the training set, which consists of already retired units for which the retirement age is known. We illustrate this situation in Table 2.

Hence, including COD values in the analysis would create a false effect, causing the machine-learning model to interpret historical trends as indicating that the earlier the COD year (i.e., the older the plant) the older the retirement age. Conversely, a later COD year (i.e., a younger plant) would be interpreted as inducing a younger retirement age. We illustrate this situation in Figure 6. The bottom of Figure 2 plots the average retirement ages for all retired units. This indicates a declining average retirement age over time if units are plotted according to COD years. This situation arises because in the last three decades, there are more units still in operation than have retired (Figure 6A). Because the machine-learning model learns only from retired and not still operating units, it would conclude from this trend that older units with an earlier COD year are more likely to retire at a young age (Figure 6B).

Sample construction: training set

In the second step, we built a sample of all CFPP units retired worldwide between 2010 and 2020 using data from Global Energy Monitor.[4] This contains 1,697 CFPPs units retired in 34 countries. This sample provides our "training set" (see Table S2 and Figure S2 for a statistical description).

CellPress
OPEN ACCESS

Table 2. Illustration of reason for excluding plant age as a variable input to the retirement forecasting model

Training set			Model application set			
Country	Unit	Retirement age, years	Country	Unit	COD	Retirement age, years
Australia	Kwinana-A power station Unit 1	40	Australia	Bayswater power station Unit 1	1985	?
Australia	Swanbank-B power station Unit 2	40	Australia	Bluewaters power station Unit 1	2009	?
Australia	Collinsville power station Unit 5	38	Australia	Collie power station Unit 1	1999	?

Data are from Global Energy Monitor.[4]

We selected the period 2010–2020 because of the highest number of retirements (over 1,800 units) compared with preceding decadal periods (for example, the period 2000–2009 saw the retirement of only around 600 units) (Table S2; Figure S2). Moreover, this period has more data available for the factors influencing retirement.

When building the sample, we excluded countries for which data were unavailable. We also treated units retired before 10 years as outliers, excluding these from the analysis. These outliers totaled 53 or 3% of all units, thus comprising an insignificant portion of our sample. We made this decision after observing that the concerned units are almost all concentrated in China, and that particular circumstances were frequently behind these extreme cases of early retirement (Table S3). For instance, many plants were prematurely shut down due to government orders after legal violations, financial difficulties in the power utility, or as part of short-term demonstration projects.

We divided the sample into three groups: China, the United States, and the ROW. This strategy deals with the influence of China and the United States, which collectively represent 1,100 of 1,697 units. We thus prevent the unique factors influencing historical retirements in China and the United States (such factors would include the strong influence of government in China in closing CFFPs and the shift from coal to gas in the USA enabled by shale gas production) from affecting other countries' results, increasing the model's forecasting accuracy. When making this decision, we compared the accuracy of results between a single sample for the entire world and the aforementioned three regional groups. Although both models successfully recreated the historical retirement trend with high accuracy, the retirement age has a slightly different result (see Table S4). Concretely, a single model resulted in unclear correlations for the most influential factors based on the SHAP analysis. Therefore, we decided to use three separate models for China, the United States, and the ROW.

Sample construction: Model application set

In the third step, we again leveraged data from the Global Energy Monitor to build a sample of 6,541 operating and under-construction units (the sample excludes units classified by Global Energy Monitor as permitted, pre-permitted or announced) in 66 countries (henceforth the "model application" set). We then apply the model developed with the previous training set to forecast the future retirement age of each CFPP unit in this dataset (see Forecasting retirement ages using supervised machine learning).

The discrepancy between the countries in the training model and model application set is unavoidable. The countries in the training model cover those that have already experienced CFPP retirement and for which data are available. Conversely, the model application set is composed of all countries with operating or planned CFPP units and with data available.

In addition, we excluded units with a planned commercial operation beyond 2025 due to a high risk of cancellation. Indeed, the year 2021 alone saw the cancellation of 2,688 CFPP units previously under planning.[4] Furthermore, cancellations are increasing as governments and societal investors increasingly balk at the high carbon emissions of coal power and elevated costs relative to renewables, which are rapidly plummeting.

Data sources for influencing factors

In the fourth step, to measure the influence of factors described by the literature as contributing to plant retirements, we leveraged various data sources listed in Table 1 for each country in our sample. The plant-level factor (F-1) comes from Global Energy Monitor,[4] whereas country-level data were retrieved from several sources, such as the World Bank (2021)[36] and BP Statistical Review.[28] When integrating country-level data, we ensured that values correspond with each unit's retirement year. For example, unit A that retired in 2019 would be paired with GDP data in 2019. However, our forecasting analysis (the model application set) uses only data from 2020, the latest year with data available.

Forecasting retirement ages using supervised machine learning

In the fifth step, we employed supervised machine learning to identify the factors influencing historical retirement ages as described in the following subsections.

Random forest regression

We adopted Random Forest Regression for the regression forecasting using machine learning because of its quick training time and acceptable error balance.[60] This method's regressive capacity has been widely demonstrated in various disciplines, including biological medicine,[61] environmental monitoring,[62] and astronomy.[63] Although there are cases where the "neuron" concept in neural network methods is superior to the "tree" concept, the Random Forest Regression offers several advantages that better suited our data. These especially offer greater ease and speed when determining the model structure and during its application and tuning.[64] For energy-related forecasting, Alova et al.[65] used a similar supervised machine-learning technique. However, their binary output prevented us from directly appropriating the same model in our study, which forecasts retirement ages.

We ran the entire analysis with Python through *Google Colaboratory*. The entire script is accessible from Zenodo.[59]

Hyperparameter tuning and accuracy testing

When developing the Random Forest Regression, we used forecasting accuracy as the main parameter to determine the sufficiency of the model. The best settings for our model were identified by running hyperparameter tuning, where we tuned the model's hyperparameters to increase forecasting performance and to avoid overfitting. This removed patterns deemed insignificant for forecasting or that do not generalize beyond the training sample for core and learning control parameters. Results for hyperparameter tuning and accuracy testing appear in Tables S4–S6. Here we show that accuracy results for China, the United States, and the ROW are 82.13%, 78.79%, and 78.53%, respectively. Because reasonable forecasts can be made with a value of 50%, our three models indicate the ability to make reliable forecasts.[66]

Feature selection (SHAP value)

To identify each factor's contribution to a unit's retirement, we used SHAP values, informed by prior literature that recognizes the consistency and accuracy of these values for model forecasting.[67] SHAP values, given in log odds, reflect a factor's influence on retirement age forecasting. A positive SHAP value indicates that a factor extends a unit's lifetime operation, whereas a negative value shortens it.

Lifetime operational CO_2 emissions

In the sixth step, we calculate the lifetime CO_2 emissions expected to occur from each unit in light of forecasted retirement ages. We set the base year to 2025, the last planned year of commercial operation for units still under construction. Lifetime operational emissions are compared using two scenarios: first, remaining lifetime emissions based on the average lifetime operation of units retired between 2010 and 2020[4]; and second, remaining lifetime emissions based on our retirement forecasting results. Emissions are calculated as

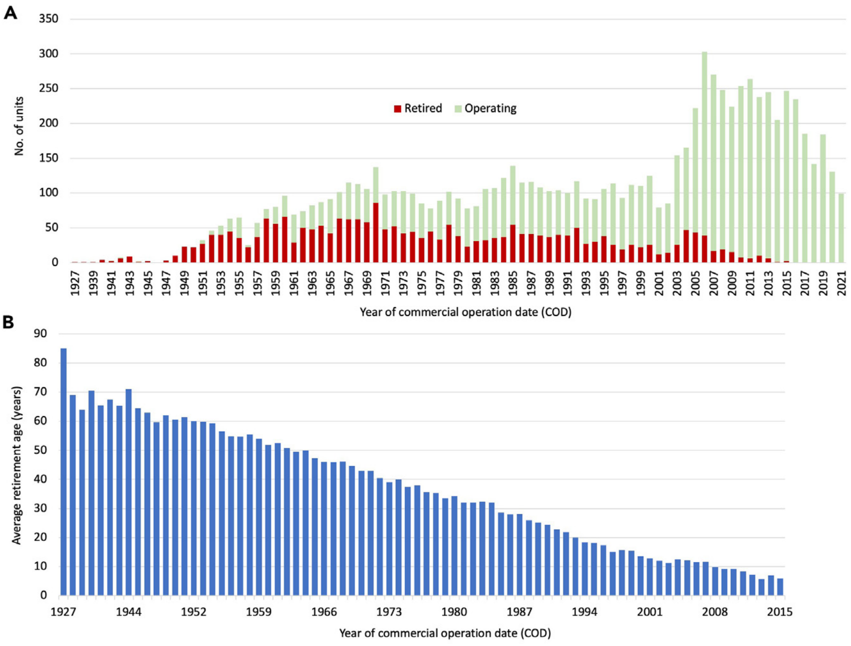

Figure 6. The false effect on retirement ages if including COD values in the historical analysis

These figures show the attributes of all units retired or operating (A: n = 8,925; B: n = 2,437) between 1927 and 2021 of its operating year (COD).

$$\text{Lifetime operation } CO_2 \text{ emissions} = \text{remaining lifetime of unit} \times \text{annual } CO_2,$$

where remaining lifetime is COD − retirement age, and annual CO_2 is annual emissions of unit (sourced from Global Energy Monitor[4]).

Coal lock-in index

We estimate the degree of lock-in each country is expected to face to coal power based on three components known to enable or hamper efforts to reduce dependence on coal: (1) the age of a country's CFPP fleet, (2) the share of coal in the power mix, and (3) total installed capacity. Shown in the second formula, our calculation of age captures the remaining operational lifetime of a unit (as of 2025) weighted by the capacity of a country's CFFP fleet. We extend on the carbon lock-in index proposed by Neofytou et al.[56] by integrating the forecasted retirement age into the model in place of a flat 40 years. In line with these authors, the index focuses on the consumption side of coal. We use the root square to reduce the range of the index. The calculation is as follows:

$$\text{Coal lockin} = \sqrt{\text{age} * \text{coal share}},$$

where coal share represents share of coal (%) in the electricity mix in 2025 (to obtain the share of coal power in 2025, since the latest year for which available is 2020, we follow Alova et al.[65] by extrapolating data from 2015 to 20 for the period 2021–2025 using the 3-year moving average. However, this forecasting method could not work for a country with a 0% share of coal power in 2020. Thus, for countries with no coal power in 2020 but with units under construction and that are expected to come online during 2021–2025, we assume a 1% coal share in 2025. These countries are Honduras, Senegal, Tajikistan, and United Arab Emirates (4 out of 66 countries) (base year), and age represents CFPP fleet age weighted by capacity, based on Neofytou et al.[56] as below, and set to base year 2025:

$$\text{Age} = \frac{\sum_{i=1}^{n} Cap_i[(\text{commercial operation date}+\text{retirement age prediction}) - \text{base year})]}{\sum_{i=1}^{n} Cap_i},$$

where cap represents capacity of all CFPP units (sourced from Global Energy Monitor), COD represents year plant operation commenced (sourced from Global Energy Monitor), retirement age forecasting represents result from the machine-learning analysis, and the base year is 2025.

SUPPLEMENTAL INFORMATION

Supplemental information can be found online at https://doi.org/10.1016/j.patter.2023.100776.

ACKNOWLEDGMENTS

G.T. acknowledges support from Kaken funds from the Japan Society for the Promotion of Science and Technology (21H04941). N.M. gratefully acknowledges ideological and financial support from the Heinrich Böll Foundation. We finally would like to thank anonymous reviewers for their helpful comments.

AUTHOR CONTRIBUTIONS

A.E. is the lead author. All authors conceptualized the study. A.E. acquired the data, conducted the analysis, and produced all figures and tables. A.E. and G.T. wrote the original draft. G.T. and N.M. provided feedback on all previous working steps. A.E., G.T., N.M., and K.M. discussed and interpreted the results and contributed to the manuscript.

DECLARATION OF INTERESTS

The authors declare no competing interests.

CellPress
OPEN ACCESS

Received: March 19, 2023
Revised: March 24, 2023
Accepted: May 19, 2023
Published: June 21, 2023

REFERENCES

1. IEA (2022). Electricity Market Report.
2. Jewell, J., Vinichenko, V., Nacke, L., and Cherp, A. (2019). Prospects for powering past coal. Nat. Clim. Chang. *9*, 592–597. https://doi.org/10.1038/s41558-019-0509-6.
3. Casey, J.A., Su, J.G., Henneman, L.R.F., Zigler, C., Neophytou, A.M., Catalano, R., Gondalia, R., Chen, Y.T., Kaye, L., Moyer, S.S., et al. (2020). Coal-fired power plant closures and retrofits reduce asthma morbidity in the local population. Nat. Energy *5*, 398–408. https://doi.org/10.1038/s41560-020-0600-2.
4. Global Energy Monitor (2022). Global Coal Plant Tracker.
5. Farfan, J., and Breyer, C. (2017). Structural changes of global power generation capacity towards sustainability and the risk of stranded investments supported by a sustainability indicator. J. Clean. Prod. *141*, 370–384. https://doi.org/10.1016/j.jclepro.2016.09.068.
6. Shearer, C., Fofrich, R., and Davis, S.J. (2017). Future CO2 emissions and electricity generation from proposed coal-fired power plants in India. Earth's Futur *5*, 408–416. https://doi.org/10.1002/2017EF000542.
7. Erickson, P., Kartha, S., Lazarus, M., and Tempest, K. (2015). Assessing carbon lock-in. Environ. Res. Lett. *10*, 084023. https://doi.org/10.1088/1748-9326/10/8/084023.
8. IPCC (2022). Mitigation of Climate Change Summary for Policymakers (SPM) (Cambridge Univ. Press), pp. 1–30.
9. Koplitz, S.N., Jacob, D.J., Sulprizio, M.P., Myllyvirta, L., and Reid, C. (2017). Burden of disease from rising coal-fired power plant emissions in southeast Asia. Environ. Sci. Technol. *51*, 1467–1476. https://doi.org/10.1021/acs.est.6b03731.
10. Littlecott, C., Roberts, L., Şenlen, Ö., Burton, J., Joshi, M., Shearer, C., and Ewen, M. (2021). No New Coal by 2021: The Collapse of the Global Coal Pipeline.
11. Edenhofer, O., Steckel, J.C., Jakob, M., and Bertram, C. (2018). Reports of coal's terminal decline may be exaggerated. Environ. Res. Lett. *13*, 024019. https://doi.org/10.1088/1748-9326/aaa3a2.
12. Steckel, J.C., and Jakob, M. (2021). The political economy of coal: lessons learnt from 15 country case studies. World Dev. Perspect. *24*, 100368. https://doi.org/10.1016/j.wdp.2021.100368.
13. Jakob, M., and Steckel, J.C. (2022). The Political Economy of Coal (Routledge). https://doi.org/10.1016/0301-4215(86)90099-6.
14. Diluiso, F., Walk, P., Manych, N., Cerutti, N., Chipiga, V., Workman, A., Ayas, C., Cui, R.Y., Cui, D., Song, K., et al. (2021). Coal transitions - Part 1: a systematic map and review of case study learnings from regional, national, and local coal phase-out experiences. Environ. Res. Lett. *16*, 113003. https://doi.org/10.1088/1748-9326/ac1b58.
15. UNFCCC (2016). Just Transition of the Workforce and the Creation of Decent Work and Quality Jobs. Technical paper.
16. Manych, N., and Jakob, M. (2021). Why coal? – The political economy of the electricity sector in the Philippines. Energy Sustain. Dev. *62*, 113–125. https://doi.org/10.1016/j.esd.2021.03.012.
17. Caldecott, B., Clark, A., Koskelo, K., Mulholland, E., and Hickey, C. (2021). Stranded assets: environmental drivers, societal challenges, and supervisory responses. Annu. Rev. Environ. Resour. *46*, 417–447. https://doi.org/10.1146/annurev-environ-012220-101430.
18. Sen, S., and Ganguly, S. (2017). Opportunities, barriers and issues with renewable energy development – a discussion. Renew. Sustain. Energy Rev. *69*, 1170–1181. https://doi.org/10.1016/j.rser.2016.09.137.
19. Shukla, A.K., Sudhakar, K., and Baredar, P. (2017). Renewable energy resources in South Asian countries: challenges, policy and recommendations. Resour. Technol. *3*, 342–346. https://doi.org/10.1016/j.reffit.2016.12.003.
20. Trencher, G., Downie, C., Hasegawa, K., and Asuka, J. (2020). Divestment trends in Japan's international coal businesses. Renew. Sustain. Energy Rev. *124*, 109779. https://doi.org/10.1016/j.rser.2020.109779.
21. Kefford, B.M., Ballinger, B., Schmeda-Lopez, D.R., Greig, C., and Smart, S. (2018). The early retirement challenge for fossil fuel power plants in deep decarbonisation scenarios. Energy Pol. *119*, 294–306. https://doi.org/10.1016/j.enpol.2018.04.018.
22. Oberschelp, C., Pfister, S., Raptis, C.E., and Hellweg, S. (2019). Global emission hotspots of coal power generation. Nat. Sustain. *2*, 113–121. https://doi.org/10.1038/s41893-019-0221-6.
23. Maamoun, N., Kennedy, R., Jin, X., and Urpelainen, J. (2020). Identifying coal-fired power plants for early retirement. Renew. Sustain. Energy Rev. *126*, 109833. https://doi.org/10.1016/j.rser.2020.109833.
24. Cui, R.Y., Hultman, N., Edwards, M.R., He, L., Sen, A., Surana, K., McJeon, H., Iyer, G., Patel, P., Yu, S., et al. (2019). Quantifying operational lifetimes for coal power plants under the Paris goals. Nat. Commun. *10*. 4759–9. https://doi.org/10.1038/s41467-019-12618-3.
25. Cui, R.Y., Hultman, N., Cui, D., McJeon, H., Yu, S., Edwards, M.R., Sen, A., Song, K., Bowman, C., Clarke, L., et al. (2021). A plant-by-plant strategy for high-ambition coal power phaseout in China. Nat. Commun. *12*, 1468. https://doi.org/10.1038/s41467-021-21786-0.
26. Brutschin, E., Schenuit, F., Van Ruijven, B., and Riahi, K. (2022). Exploring enablers for an ambitious coal phaseout. PaG. *10*, 200–212.
27. World Bank (2018). Carbon Price (World Bank), pp. 50–51. https://doi.org/10.4324/9781315270326-31.
28. BP (2021). BP Statistical Review 2021.
29. EMBER (2021). Global Electricity Review 2021.
30. Trencher, G., Healy, N., Hasegawa, K., and Asuka, J. (2019). Discursive resistance to phasing out coal-fired electricity: narratives in Japan's coal regime. Energy Pol. *132*, 782–796. https://doi.org/10.1016/j.enpol.2019.06.020.
31. Hughes, J.P. (2016). Regulatory treatment of uneconomic power plants. Electr. J. *29*, 28–32. https://doi.org/10.1016/j.tej.2016.07.008.
32. Mo, J., Cui, L., and Duan, H. (2021). Quantifying the implied risk for newly-built coal plant to become stranded asset by carbon pricing. Energy Econ. *99*, 105286. https://doi.org/10.1016/j.eneco.2021.105286.
33. Pahle, M. (2010). Germany's dash for coal: exploring drivers and factors. Energy Pol. *38*, 3431–3442. https://doi.org/10.1016/j.enpol.2010.02.017.
34. Ross, M.T. (2018). The future of the electricity industry: implications of trends and taxes. Energy Econ. *73*, 393–409. https://doi.org/10.1016/j.eneco.2018.03.022.
35. Mo, J., Zhang, W., Tu, Q., Yuan, J., Duan, H., Fan, Y., Pan, J., Zhang, J., and Meng, Z. (2021). The role of national carbon pricing in phasing out China's coal power. iScience *24*, 102655. https://doi.org/10.1016/j.isci.2021.102655.
36. World Bank (2021d). Environmental, Social and Governance (ESG).
37. World Bank (2021b). Price of Electricity (Nimble fins).
38. Scholvin, S. (2014). South Africa's energy policy: constrained by nature and path dependency. J. South Afr. Stud. *40*, 185–202. https://doi.org/10.1080/03057070.2014.889361.
39. Tan, H., Thurbon, E., and Sung-Young Kim, J.A.M. (2021). Overcoming incumbent resistance to the clean energy shift, How local government act as change agents in coal power station closure in China.pdf. Energy Policy.
40. World Bank (2021c). Regulatory Indicators for Sustainable Energy. https://rise.esmap.org/.
41. Gallagher, K.S., Bhandary, R., Narassimhan, E., and Nguyen, Q.T. (2021). Banking on coal? Drivers of demand for Chinese overseas investments in coal in Bangladesh, India, Indonesia and Vietnam. Energy Res. Social Sci. *71*, 101827. https://doi.org/10.1016/j.erss.2020.101827.

42. Wendling, Z.A., Emerson, J.W., de Sherbinin, A., Esty, D.C., et al. (2020). Environmental Performance Index (New Haven, CT: Yale Center for Environmental Law & Policy. epi.yale.edu).
43. Wang, D., Xue, X., and Wang, Y. (2021). Overcapacity risk of China's coal power industry: a comprehensive assessment and driving factors. Sustain. Times *13*, 1426. https://doi.org/10.3390/su13031426.
44. Dorband, I.I., Jakob, M., and Steckel, J.C. (2020). Unraveling the political economy of coal: insights from Vietnam. Energy Pol. *147*, 111860. https://doi.org/10.1016/j.enpol.2020.111860.
45. World Bank (2021a). World Development Indicators.
46. Blondeel, M., Van de Graaf, T., and Haesebrouck, T. (2020). Moving beyond coal: exploring and explaining the powering past coal alliance. Energy Res. Social Sci. *59*, 101304. https://doi.org/10.1016/j.erss.2019.101304.
47. Fell, H., and Kaffine, D.T. (2018). The fall of coal: joint impacts of fuel prices and renewables on generation and emissions. Am. Econ. J. Econ. Pol. *10*, 90–116. https://doi.org/10.1257/pol.20150321.
48. Gray, D., and Bernell, D. (2020). Tree-hugging utilities? The politics of phasing out coal and the unusual alliance that passed Oregon's clean energy transition law. Energy Res. Social Sci. *59*, 101288. https://doi.org/10.1016/J.ERSS.2019.101288.
49. IRENA (2021). Statistical Profile.
50. Dodd, T., and Nelson, T. (2019). Trials and tribulations of market responses to climate change: insight through the transformation of the Australian electricity market. Aust. J. Manag. *44*, 614–631. https://doi.org/10.1177/0312896219874096.
51. Trencher, G., Rinscheid, A., Duygan, M., Truong, N., and Asuka, J. (2020). Revisiting carbon lock-in in energy systems: explaining the perpetuation of coal power in Japan. Energy Res. Social Sci. *69*, 101770. https://doi.org/10.1016/j.erss.2020.101770.
52. Webb, J., de Silva, H.N., and Wilson, C. (2020). The future of coal and renewable power generation in Australia: a review of market trends. Econ. Anal. Pol. *68*, 363–378. https://doi.org/10.1016/j.eap.2020.10.003.
53. Fell, H., and Kaffine, D.T. (2014). A One-Two Punch: Joint Effects of Natural Gas Abundance and Renewables on Coal-Fired Power Plants.
54. Tong, D., Zhang, Q., Liu, F., Geng, G., Zheng, Y., and Xue, T. (2018). Energy and the Environment Current emissions and future mitigation pathways of coal-fired power plants in China from 2010 to 2030 Current emissions and future mitigation pathways of coal-fired. Environ. Sci. Technol. *52*, 12905–12914. https://doi.org/10.1021/acs.est.8b02919.
55. Friedlingstein, P., Jones, M.W., Sullivan, M.O., Andrew, R.M., Bakker, D.C.E., Hauck, J., Quéré, C.L., Peters, G.P., and Peters, W. (2022). Global Carbon Budget 2021 (Earth Syst. Sci. Data), pp. 1917–2005. https://doi.org/10.5194/essd-14-1917-2022.
56. Neofytou, H., Nikas, A., and Doukas, H. (2020). Sustainable energy transition readiness: a multicriteria assessment index. Renew. Sustain. Energy Rev. *131*, 109988. https://doi.org/10.1016/j.rser.2020.109988.
57. Steckel, J., and Jakob, M. (2022). To End Coal, Adapt to Regional Realities.
58. Muttitt, G., Price, J., Pye, S., and Welsby, D. (2023). Socio-political feasibility of coal power phase-out and its role in mitigation pathways. Nat. Clim. Chang. *13*, 140–147. https://doi.org/10.1038/s41558-022-01576-2.
59. Edianto, A., Trencher, G., Manych, N., and Matsubae, K. (2023). Phase-out Challenges for Coal Power: Predicting Retirement Ages and Lock-In from Historical Data with Random Forest Regression. https://doi.org/10.5281/ZENODO.7765303.
60. Li, C., Tao, Y., Ao, W., Yang, S., and Bai, Y. (2018). Improving forecasting accuracy of daily enterprise electricity consumption using a random forest based on ensemble empirical mode decomposition. Energy *165*, 1220–1227. https://doi.org/10.1016/j.energy.2018.10.113.
61. Rahman, R., Dhruba, S.R., Ghosh, S., and Pal, R. (2019). Functional random forest with applications in dose-response predictions. Sci. Rep. *9*, 1628. https://doi.org/10.1038/s41598-018-38231-w.
62. Tan, K., Wang, H., Chen, L., Du, Q., Du, P., and Pan, C. (2020). Estimation of the spatial distribution of heavy metal in agricultural soils using airborne hyperspectral imaging and random forest. J. Hazard Mater. *382*, 120987. https://doi.org/10.1016/j.jhazmat.2019.120987.
63. Vasylenko, M., and Dobrycheva, D. (2019). Machine learning for the extragalactic astronomy educational manual. Proc. Int. Astron. Union *15*, 461–463. https://doi.org/10.1017/S1743921321000132.
64. Liu, J., Huang, Q., Ulishney, C., and Dumitrescu, C.E. (2022). Comparison of random forest and neural network in modeling the performance and emissions of a natural gas spark ignition engine. J. Energy Resour. Technol. *144*, 1–12. https://doi.org/10.1115/1.4053301.
65. Alova, G., Trotter, P.A., and Money, A. (2021). A machine-learning approach to predicting Africa's electricity mix based on planned power plants and their chances of success. Nat. Energy *6*, 158–166. https://doi.org/10.1038/s41560-020-00755-9.
66. Kasemset, C., Sae-Haew, N., and Sopadang, A. (2014). Multiple regression model for forecasting quantity of supply of off-season longan. Chiang Mai Univ. J. Nat. Sci. *13*, 391–402. https://doi.org/10.12982/cmujns.2014.0044.
67. Sayres, R., Taly, A., Rahimy, E., Blumer, K., Coz, D., Hammel, N., Krause, J., Narayanaswamy, A., Rastegar, Z., Wu, D., et al. (2017). Using a deep learning algorithm and integrated gradients explanation to assist grading for diabetic retinopathy. In 31st Conference on Neural Information Processing Systems (NIPS), pp. 552–564. https://doi.org/10.1016/j.ophtha.2018.11.016.

Patterns, Volume *4*

Supplemental information

Forecasting coal power plant retirement ages and lock-in with random forest regression

Achmed Edianto, Gregory Trencher, Niccolò Manych, and Kazuyo Matsubae

Supplemental Information for

Forecasting coal power plant retirement ages and lock-in with random forest regression

Achmed Edianto, Gregory Trencher, Niccolò Manych, Kazuyo Matsubae

Supplemental graph for global coal-fired power plant trends

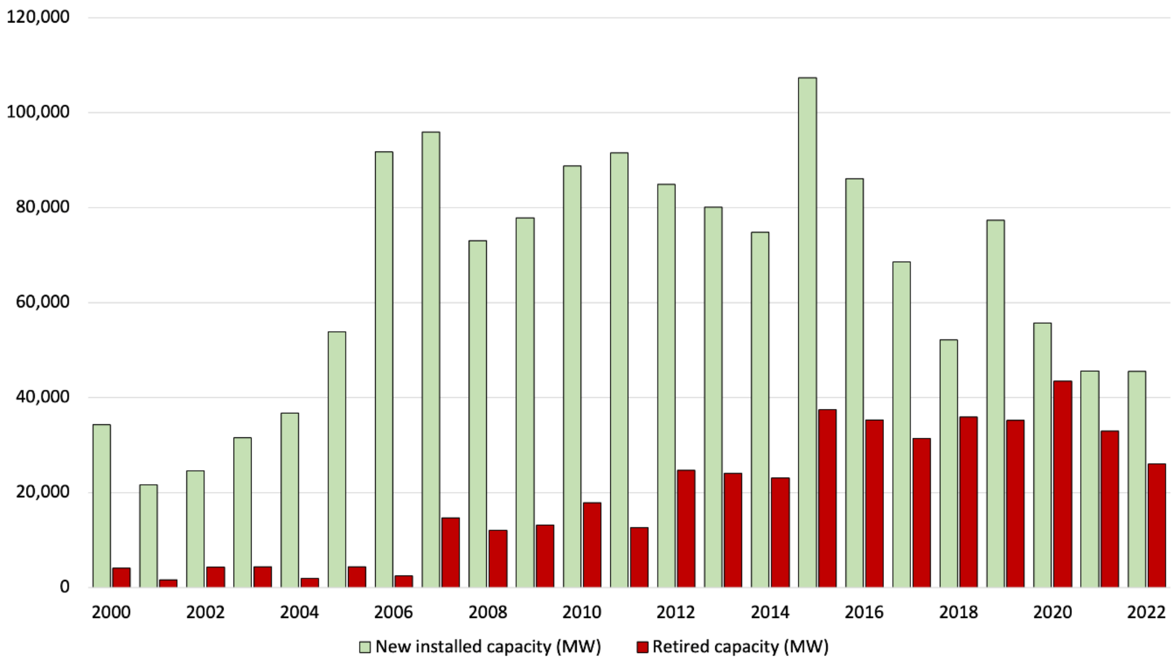

Figure S1. New installed capacity (MW) vs retired capacity (MW) (Global Energy Monitor 2022)

Table S1. Status of coal-fired power plants by country (as of 2022)

Country	Announced + Pre-permit + Permitted		Construction		Operating		Retired 2000-2022	
	MW	Unit	MW	Unit	MW	Unit	MW	Unit
Albania	0	0	0	0	0	0	0	0
Argentina	0	0	120	1	495	2	0	0
Australia	1,000	1	0	0	23,977	57	7,107	50
Austria	0	0	0	0	0	0	1,993	10
Bangladesh	6,700	7	5,724	9	2,855	7	0	0
Belarus	0	0	0	0	0	0	0	0
Belgium	0	0	0	0	0	0	2,865	19
Bosnia and Herzegovina	1,350	4	0	0	2,073	10	0	0
Botswana	450	2	0	0	732	8	0	0
Brazil	1,666	5	0	0	3,177	17	518	5
Brunei	0	0	0	0	220	4	0	0
Bulgaria	0	0	0	0	4,709	30	1,380	8
Cambodia	700	2	315	3	1,405	9	0	0
Canada	0	0	0	0	4,707	19	14,818	47
Chile	0	0	0	0	4,323	20	1,193	8
China	250,069	400	115,472	211	1,092,889	3,092	119,268	1,087
Colombia	0	0	0	0	1,646	14	0	0
Côte d'Ivoire	0	0	0	0	0	0	0	0
Croatia	0	0	0	0	210	1	0	0
Czech Republic	0	0	0	0	7,445	69	2,997	18
Denmark	0	0	0	0	1,560	4	3,443	14
Djibouti	0	0	0	0	0	0	0	0
Dominican Republic	0	0	0	0	1,064	5	0	0
DR Congo	0	0	0	0	0	0	0	0
Egypt	0	0	0	0	0	0	0	0
El Salvador	0	0	0	0	0	0	0	0
Eswatini	300	3	0	0	0	0	0	0
Ethiopia	0	0	0	0	0	0	0	0
Finland	0	0	0	0	1,468	8	2,319	15
France	0	0	0	0	2,507	11	6,619	23
Georgia	0	0	0	0	0	0	0	0
Germany	0	0	0	0	40,505	102	25,181	122
Ghana	0	0	0	0	0	0	0	0
Greece	0	0	660	1	2,225	7	3,053	14
Guadeloupe	0	0	0	0	64	2	38	1
Guatemala	0	0	0	0	1,172	16	0	0
Guinea	0	0	0	0	0	0	0	0
Honduras	0	0	0	0	105	3	0	0
Hong Kong	0	0	0	0	6,110	14	500	2
Hungary	0	0	0	0	944	7	515	10

India	28,503	44	32,000	49	234,256	840	15,651	158
Indonesia	7,480	30	18,849	60	40,647	231	0	0
Iran	0	0	650	2	0	0	0	0
Ireland	0	0	0	0	915	3	0	0
Israel	0	0	0	0	4,325	9	575	1
Italy	0	0	0	0	6,166	14	4,194	21
Jamaica	0	0	0	0	0	0	0	0
Japan	500	1	2,450	4	52,978	152	2,674	19
Kazakhstan	756	2	65	1	13,033	95	210	4
Kenya	64	1	0	0	0	0	0	0
Kosovo	0	0	0	0	1,290	5	190	2
Kyrgyzstan	600	1	0	0	826	9	195	3
Laos	7,036	12	0	0	1,878	3	0	0
Latvia	0	0	0	0	0	0	0	0
Madagascar	30	1	0	0	120	3	0	0
Malawi	400	7	0	0	0	0	0	0
Malaysia	0	0	0	0	13,280	25	0	0
Mauritius	0	0	0	0	195	5	0	0
Mexico	0	0	0	0	5,378	15	0	0
Moldova	0	0	0	0	0	0	0	0
Mongolia	7,030	10	50	1	960	11	0	0
Montenegro	0	0	0	0	225	1	0	0
Morocco	0	0	0	0	4,257	14	0	0
Mozambique	1,200	6	0	0	0	0	0	0
Myanmar	0	0	0	0	190	4	0	0
Namibia	0	0	0	0	120	4	0	0
Netherlands	0	0	0	0	4,152	5	3,665	6
New Zealand	0	0	0	0	500	2	500	2
Niger	200	4	0	0	0	0	0	0
Nigeria	0	0	0	0	285	7	0	0
North Korea	0	0	0	0	3,250	45	0	0
North Macedonia	0	0	0	0	800	4	0	0
Oman	0	0	0	0	0	0	0	0
Pakistan	4,010	8	732	3	7,638	20	0	0
Panama	0	0	0	0	306	2	120	3
Papua New Guinea	0	0	0	0	0	0	0	0
Peru	0	0	0	0	0	0	135	1
Philippines	1,620	9	735	5	11,893	58	157	3
Poland	0	0	100	1	29,130	145	7,124	60
Portugal	0	0	0	0	0	0	2,028	7
Romania	0	0	0	0	2,955	14	4,450	34
Russia	3,173	12	70	2	39,925	312	8,301	62
Senegal	0	0	0	0	155	2	0	0
Serbia	1,350	4	350	1	4,405	17	32	1
Slovakia	0	0	0	0	769	7	724	8

Slovenia	0	0	0	0	1,069	5	535	5
South Africa	1,635	2	1,600	2	43,624	90	1,180	15
South Korea	0	0	3,140	3	39,154	80	3,420	11
Spain	0	0	0	0	2,210	8	10,892	40
Sri Lanka	0	0	0	0	900	3	0	0
Sudan	0	0	0	0	0	0	0	0
Sweden	0	0	0	0	0	0	291	3
Syria	0	0	0	0	0	0	0	0
Taiwan	0	0	0	0	19,244	55	2,234	14
Tajikistan	0	0	0	0	400	4	0	0
Tanzania	600	4	0	0	0	0	0	0
Thailand	600	1	0	0	6,138	21	675	6
Türkiye	10,438	22	145	1	20,093	77	360	1
Ukraine	660	2	0	0	9,320	56	3,821	13
United Arab Emirates	0	0	0	0	0	0	1,200	2
United Kingdom	0	0	0	0	4,140	8	30,623	82
United States	0	0	0	0	212,042	441	148,514	738
Uzbekistan	600	2	0	0	2,493	12	241	4
Venezuela	0	0	0	0	0	0	0	0
Vietnam	1,210	6	6,120	10	24,637	73	0	0
Zambia	300	2	0	0	330	3	0	0
Zimbabwe	4,570	17	940	4	1,000	8	0	0
**Total**	346,800	634	190,287	374	2,082,581	6,565	448,718	2,782

Note S1. Procedure for literature review

Consisting of three segments, the string essentially sets our core topic (coal power, coal plant, etc.), the act of retirement or construction (closure, shutdown, development etc.) and influencing conditions (influence, driver etc.). This was iteratively developed through a procedure where we set search terms, verified the accuracy and coverage of hits, identified other important search terms from results and then, finally, fine-tuned the search string until an acceptable level was achieved.

The search was carried out on October 2021 with the following conditions:

- Publication type: Articles and reviews
- Publication language: English
- Search scope: Title, author keywords, abstract
- Temporal scope: All papers published up to October 31, 2021
- Excluded academic fields: Agriculture, biochemical, chemical, chemical engineering, computer science, earth science, engineering, immunology, mathematic, medicine, material science, neuroscience, nursing, pharmacy, physics.

The search string, in Scopus language, appears as follows. We set wild cards (*) to ensure that synonyms for CFPP closure and retirement were found independently from word endings:

TITLE-ABS-KEY ("coal power" OR "coal generation" OR "coal electricity" OR "coal-fired" OR "coal unit" OR "coal plant") AND TITLE-ABS-KEY ("clos*" OR "closure" OR "phase-out" OR "phasing-out" OR "shut-down" OR "retir*" OR "construct" OR "develop" OR "build" OR "decline" OR "stranded" OR "stranding") AND TITLE-ABS-KEY ("factor" OR "influence" OR "indicator" OR "condition" OR "driver" OR "barrier") AND DOCTYPE (ar OR re) AND

PUBYEAR > 2000 AND (EXCLUDE (SUBJAREA , "ENGI") OR EXCLUDE (SUBJAREA , "CENG") OR EXCLUDE (SUBJAREA , "CHEM") OR EXCLUDE (SUBJAREA , "EART") OR EXCLUDE (SUBJAREA , "MATE") OR EXCLUDE (SUBJAREA , "PHYS") OR EXCLUDE (SUBJAREA , "MATH") OR EXCLUDE (SUBJAREA , "MEDI") OR EXCLUDE (SUBJAREA , "COMP") OR EXCLUDE (SUBJAREA , "AGRI") OR EXCLUDE (SUBJAREA , "BIOC") OR EXCLUDE (SUBJAREA , "PHAR") OR EXCLUDE (SUBJAREA , "NEUR") OR EXCLUDE (SUBJAREA , "IMMU") OR EXCLUDE (SUBJAREA , "NURS")) AND (LIMIT-TO (LANGUAGE , "English"))

The above search procedure resulted in 194 potentially relevant publications to which we added further ones identified from the in-text citations, during the later stages of our review. To identify relevant publications, we set up criteria to be met by evidence contained in the title and abstract. That is, we only considered studies that:

- Explicitly mention drivers or barriers that affect coal power development, retirement or phase-out
- Explicitly analyze the influence of these factors on coal power development, retirement or phase-out

Application of these criteria led to a set of 25 potentially relevant publications. We then read the full-text versions and further excluded those deemed irrelevant. We also excluded publications for which the full text was unavailable. This procedure resulted in a final corpus of 19 publications that were subsequently examined (**Table 2 in main text**).

Data

Table S2. Sample description

	**2000-2009**		**2010-2020**	
	Units	MW	Units	MW
	357	31,872	626	80,824
**United States**	84	8,727	589	117,821
**Germany**	28	3,358	114	11,326
**United Kingdom**	28	4,342	75	15,073
**India**	20	1,881	52	25,187
**Canada**	13	2,718	42	6,011
**Belgium**	9	998	41	6,597
**Japan**	9	1,417	39	4,827
**Poland**	8	712	30	7,704
**Others**	55	6,683	229	46,298
**Total**	611	62,708	1,837	321,667

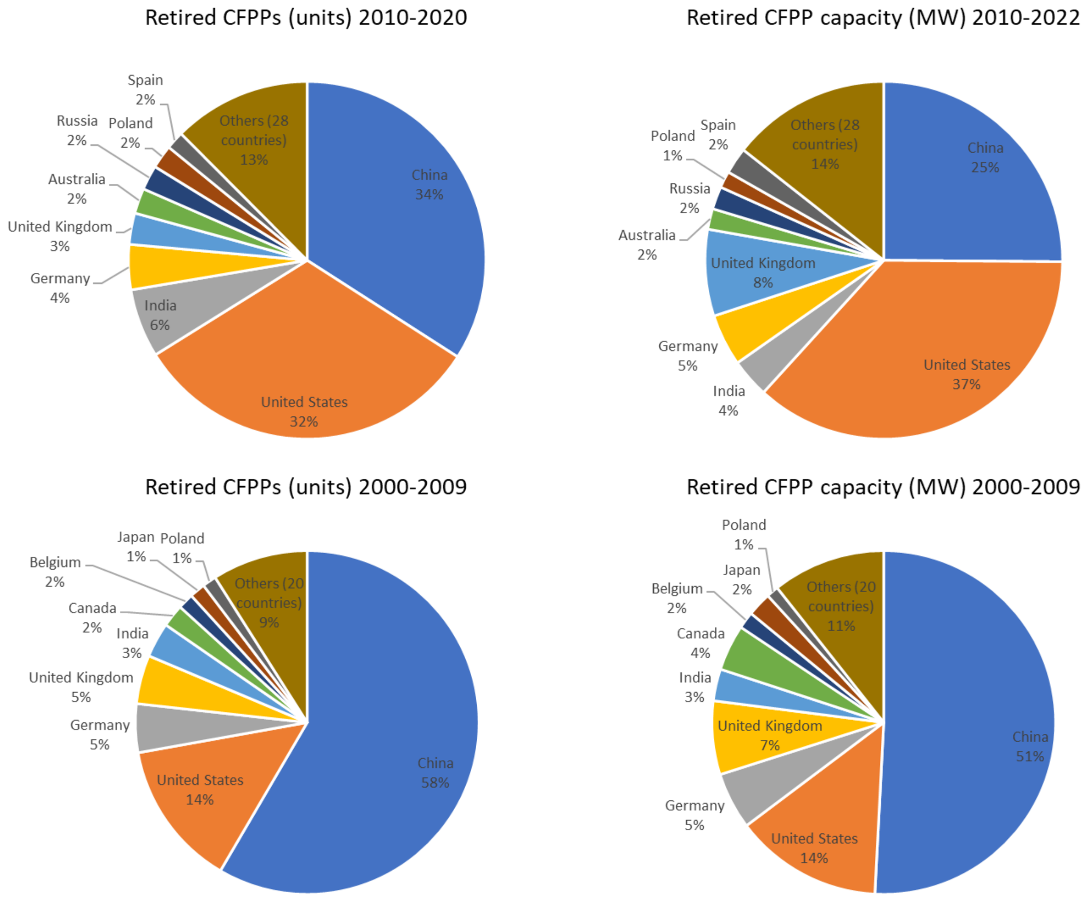

Figure S2. Distribution of retired CFPPs in historical analysis

Table S3. Retirement reasons of the outlier units (retired between 3-9 years after the start of commercial operation)

**Global Energy Monitor wiki page**	**Country**	**Subnational (province, state)**	**Unit name**	**Plant name**	**Capacity (MW)**	**Year of starting operation**	**Year of retirement**	**Retirement age (years)**	**Notes (taken from Global Energy Monitor wiki page)**
https://gem.wiki/Callide_Oxyfuel_Project	Australia	Queensland	Callide Oxyfuel Project	Callide Oxyfuel Project	30	2012	2015	3	This unit was recommissioned for a carbon capture and storage pilot project for only 3 years.
https://gem.wiki/Lianzhou_power_station	China	Guangdong	Yuelian power station Unit 3	Yuelian power station	135	2004	2013	9	The full plant was retired in 2013, after a breakout of high levels of lead in the blood of the local children.
https://gem.wiki/Lianzhou_power_station	China	Guangdong	Yuelian power station Unit 4	Yuelian power station	135	2004	2013	9	Same as above
https://gem.wiki/Guiyang_power_station	China	Guizhou	Guiyang power station Unit 4	Guiyang power station	200	2004	2013	9	This unit is part of the power plant that was founded in 1957. Since the whole power plant retired 2013, this unit need to retired although it is just operating for 9 years.
https://gem.wiki/Huadian_Shijiazhuang_power_station	China	Hebei	Huadian Shijiazhuang power station Unit 16	Huadian Shijiazhuang power station	125	2005	2014	9	After the plant was blamed for its contribution to the city's severe air pollution, this plant was forced by government authorities to retire some units, including this one.
https://gem.wiki/Yima_Gezha_power_station	China	Henan	Yima Gezha power station Unit 1	Yima Gezha power station	155	2010	2013	3	In 2013, due to the plant not meeting environmental standards, it went offline. The parent company Wuhan East Lake High Technology Group Co., Ltd is looking for buyers to take over. However, as of 2021 the plant is still offline, and is presumably retired.
https://gem.wiki/Yima_Gezha_power_station	China	Henan	Yima Gezha power station Unit 2	Yima Gezha power station	155	2009	2013	4	In 2013, due to the plant not meeting environmental standards, it went offline. The parent company Wuhan East Lake High Technology Group Co., Ltd is looking for buyers to take over. However, as of 2021 the plant is still offline, and is presumably retired.
https://gem.wiki/Henan_Zhongmai_Yong'an_power_station	China	Henan	Henan Zhongmai Yong'an power station Unit 1	Henan Zhongmai Yong'an power station	100	2005	2012	7	Based on satellite imagery, the plant appeared to stop operating in 2012

https://gem.wiki/Henan_Zhongmai_Yong'an_power_station	China	Henan	Henan Zhongmai Yong'an power station Unit 2	Henan Zhongmai Yong'an power station	100	2005	2012	7	Based on satellite imagery, the plant appeared to stop operating in 2012
https://gem.wiki/Henan_Xinwang_power_station	China	Henan	Henan Xinwang 1	Henan Xinwang power station	135	2006	2014	8	In 2014 the company, facing financial difficulty, retired the plant
https://gem.wiki/Henan_Xinwang_power_station	China	Henan	Henan Xinwang 2	Henan Xinwang power station	135	2007	2014	7	In 2014 the company, facing financial difficulty, retired the plant
https://gem.wiki/Henan_Shenhuo_power_station	China	Henan	Henan Shenhuo-2 power station Unit 2	Henan Shenhuo power station	135	2008	2017	9	No specific information on the reason for retirement provided
https://gem.wiki/Henan_Shenhuo_power_station	China	Henan	Henan Shenhuo-2 power station Unit 3	Henan Shenhuo power station	135	2008	2017	9	No specific information on the reason for retirement provided
https://gem.wiki/Hunan_Yongxing_power_station	China	Hunan	Hunan Yongxing power station Unit 1	Hunan Yongxing power station	60	2008	2014	6	No specific information on the reason for retirement provided
https://gem.wiki/Hunan_Yongxing_power_station	China	Hunan	Hunan Yongxing power station Unit 2	Hunan Yongxing power station	60	2009	2014	5	No specific information on the reason for retirement provided
https://gem.wiki/Nanjing_Huarun_Thermal_power_station	China	Jiangsu	Nanjing Huarun Thermal power station Unit 1	Nanjing Huarun Thermal power station	135	2004	2011	7	No specific information on the reason for retirement provided
https://gem.wiki/Nanjing_Huarun_Thermal_power_station	China	Jiangsu	Nanjing Huarun Thermal power station Unit 2	Nanjing Huarun Thermal power station	135	2004	2011	7	No specific information on the reason for retirement provided
https://gem.wiki/Huaneng_Suzhou_Cogen_power_station	China	Jiangsu	Huaneng Suzhou Cogen Power Station Unit 1	Huaneng Suzhou Cogen power station	60	2006	2014	8	No specific information on the reason for retirement provided
https://gem.wiki/Huaneng_Suzhou_Cogen_power_station	China	Jiangsu	Huaneng Suzhou Cogen Power Station Unit 2	Huaneng Suzhou Cogen power station	60	2006	2014	8	No specific information on the reason for retirement provided
https://gem.wiki/Nanjing-2_power_station	China	Jiangsu	Nanjing-2 Power Station Unit 3	Nanjing-2 power station	125	2008	2014	6	Part of the unit that first built in 1988. This unit follow the entire plant retirement in 2014.
https://gem.wiki/CPI_Fenyi_power_station	China	Jiangxi	Fenyi power station Unit 7	CPI Fenyi power station	100	2003	2011	8	No specific information on the reason for retirement provided

https://gem.wiki/Huaneng_Baishan_power_station	China	Jilin	Huaneng Baishan power station Unit 1	Huaneng Baishan power station	330	2011	2020	9	No specific information on the reason for retirement provided
https://gem.wiki/Huaneng_Baishan_power_station	China	Jilin	Huaneng Baishan power station Unit 2	Huaneng Baishan power station	330	2011	2020	9	No specific information on the reason for retirement provided
https://gem.wiki/Fushun_CPI_power_station	China	Liaoning	Fushun CPI power station Unit 2	Fushun CPI power station	200	2002	2011	9	No specific information on the reason for retirement provided
https://gem.wiki/Chentangzhuang_power_station	China	Tianjin	Chentangzhuang power station Unit 5	Chentangzhuang power station	135	2005	2010	5	No specific information on the reason for retirement provided
https://gem.wiki/Chentangzhuang_power_station	China	Tianjin	Chentangzhuang power station Unit 8	Chentangzhuang power station	300	2008	2015	7	No specific information on the reason for retirement provided
https://gem.wiki/Chentangzhuang_power_station	China	Tianjin	Chentangzhuang power station Unit 9	Chentangzhuang power station	300	2008	2015	7	No specific information on the reason for retirement provided
https://gem.wiki/Yulin_Jinlong_Cogen_power_station	China	Shaanxi	Yulin Jinlong Cogen power station unit 1	Yulin Jinlong Cogen power station	50	2008	2016	8	No specific information on the reason for retirement provided
https://gem.wiki/Yulin_Jinlong_Cogen_power_station	China	Shaanxi	Yulin Jinlong Cogen power station unit 2	Yulin Jinlong Cogen power station	50	2008	2016	8	No specific information on the reason for retirement provided
https://gem.wiki/Shandong_Chenming_Mill_power_station	China	Shandong	Shandong Chenming Mill power station Unit 3	Shandong Chenming Mill power station	155	2013	2018	5	No specific information on the reason for retirement provided
https://gem.wiki/Binzhou_Heating_Supply_Center_power_station	China	Shandong	Binzhou Heating Supply Center power station Unit 5	Binzhou Heating Supply Center power station	135	2013	2020	7	Plant operator Shandong Weiqiao Group was forced to close several plants and units including this one after the central government discovered the region had illegally built multiple plants.
https://gem.wiki/Binzhou_Heating_Supply_Center_power_station	China	Shandong	Binzhou Heating Supply Center power station Unit 6	Binzhou Heating Supply Center power station	135	2012	2020	8	Same as above

https://gem.wiki/Binzhou_Heating_Supply_Center_power_station	China	Shandong	Binzhou Heating Supply Center power station Unit 7	Binzhou Heating Supply Center power station	135	2012	2020	8	Same as above
https://gem.wiki/Binzhou_Heating_Supply_Center_power_station	China	Shandong	Binzhou Heating Supply Center power station Unit 8	Binzhou Heating Supply Center power station	135	2012	2020	8	Same as above
https://gem.wiki/Weiqiao_Aluminum_power_station	China	Shandong	Weiqiao Aluminum power station Unit 6-1	Weiqiao Aluminum power station	330	2012	2020	8	Same as above
https://gem.wiki/Weiqiao_Aluminum_power_station	China	Shandong	Weiqiao Aluminum power station Unit 6-2	Weiqiao Aluminum power station	330	2012	2020	8	Same as above
https://gem.wiki/Weiqiao_Aluminum_power_station	China	Shandong	Weiqiao Aluminum power station Unit 6-3	Weiqiao Aluminum power station	330	2012	2020	8	Same as above
https://gem.wiki/Weiqiao_Aluminum_power_station	China	Shandong	Weiqiao Aluminum power station Unit 6-4	Weiqiao Aluminum power station	330	2012	2020	8	Same as above
https://gem.wiki/Minjiang_power_station	China	Sichuan	Minjiang power station Unit 1	Minjiang power station	135	2006	2012	6	No specific information on the reason for retirement provided
https://gem.wiki/Minjiang_power_station	China	Sichuan	Minjiang power station Unit 2	Minjiang power station	135	2006	2012	6	No specific information on the reason for retirement provided
https://gem.wiki/Tashdian_power_station	China	Xinjiang	Tashdian power station Unit 8	Tashdian power station	125	2011	2019	8	No specific information on the reason for retirement provided

https://gem.wiki/Tashdian_power_station	China	Xinjiang	Tashdian power station Unit 9	Tashdian power station	125	2012	2019	7	No specific information on the reason for retirement provided
https://gem.wiki/Tianfu_East_power_station	China	Xinjiang	Tianfu East power station new unit 1	Tianfu East power station	135	2011	2018	7	No specific information on the reason for retirement provided
https://gem.wiki/Tianfu_East_power_station	China	Xinjiang	Tianfu East power station new unit 2	Tianfu East power station	135	2011	2018	7	No specific information on the reason for retirement provided
https://gem.wiki/Tianfu_East_power_station	China	Xinjiang	Tianfu East power station new unit 3	Tianfu East power station	135	2012	2018	6	No specific information on the reason for retirement provided
https://gem.wiki/Tianfu_West_power_station	China	Xinjiang	Tianfu West power station new Unit 1	Tianfu West power station	135	2013	2018	5	No specific information on the reason for retirement provided
https://gem.wiki/Tianfu_West_power_station	China	Xinjiang	Tianfu West power station new Unit 2	Tianfu West power station	135	2013	2018	5	No specific information on the reason for retirement provided
https://gem.wiki/Tianfu_West_power_station	China	Xinjiang	Tianfu West power station new Unit 3	Tianfu West power station	135	2013	2018	5	No specific information on the reason for retirement provided
https://gem.wiki/Urumuqi_Huanpeng_power_station	China	Xinjiang	Urumuqi Huanpeng power station Unit 3	Urumuqi Huanpeng power station	30	2009	2018	9	No specific information on the reason for retirement provided
https://gem.wiki/Urumuqi_Huanpeng_power_station	China	Xinjiang	Urumuqi Huanpeng power station Unit 4	Urumuqi Huanpeng power station	30	2009	2018	9	No specific information on the reason for retirement provided
https://gem.wiki/Albbruck_Mill_power_station	Germany	Baden-Wuerttemberg	Albbruck Mill New power station Unit 1	Albbruck Mill power station	31	2009	2012	3	No specific information on the reason for retirement provided
https://gem.wiki/Nakoso_power_station	Japan	Tōhoku	Nakoso power station Unit 10	Nakoso power station	250	2013	2020	7	This plant appears to have been retired to enable replacement with a larger and high-efficiency one
https://gem.wiki/Khabarovsk-3_power_stationa	Russia	Khabarovsk	Khabarovsk-3 power station Unit 4	Khabarovsk-3 power station	180	2006	2012	6	No specific information on the reason for retirement provided
https://gem.wiki/Sandow_power_station	United States	Texas	Sandow Station Unit 5	Sandow power station	661.5	2009	2018	9	Units 4-5 were retired in January 2018 due to economic factors including low natural gas prices and growth in renewable energy

Analysis

Table S4. Hyperparameter tuning

Groups	Hyperparameter tuning*
China	Best Parameters: {'n_estimators': 70, 'min_samples_split': 6, 'min_samples_leaf': 1, 'max_features': 'auto', 'max_depth': 100, 'bootstrap': True}
USA	Best Parameters: {'n_estimators': 90, 'min_samples_split': 6, 'min_samples_leaf': 3, 'max_features': 'auto', 'max_depth': 90, 'bootstrap': True}
Rest of the World	Best Parameters: {'n_estimators': 75, 'min_samples_split': 6, 'min_samples_leaf': 3, 'max_features': 'auto', 'max_depth': 40, 'bootstrap': True}

*Note: Best parameters from Hyperparameter tuning will always change every time we run the script code. The result appears in the table is based on the specific time when the authors run the script code.

Table S5. Accuracy results

	Model with three groups			Single model		
	China	USA	RoW	China	USA	RoW
**Average retirement age**	22	45	33	26	44	39
**Max age**	44	60	56	36	60	60
**Min age**	13	37	13	16	37	11
**Accuracy = 100 - MAPE (Mean Absolute Percentage Error)**	82.13%	78.79%	78.53%	81.30%		
**Mean Absolute Error (MAE)**	3.71	7.87	6.41	5.6		
**Mean Square Error (MSE)**	32.26	132.07	106.2	68.4		
**Root Mean Squared Error (RMSE)**	5.68	11.49	10.31	8.27		
**Notes**	SHAP value results show CO2 emissions is the most influential factor in the model, exerting a clear magnitude on early or later retirements.			Note that here the accuracy result is a single number since this is a single model. Then, we analyzed retirement ages based on the three groups (China, USA, Rest of the World) to give an equal comparison.<br><br>SHAP value results shows that GDP is the most influential factor in the model, However, the magnitude of GDP's effect on retirement is not clear.		

Note: The Mean Square Error (MSE) determines the average squared differences between the prediction and true values in the regression. Prediction models with a lower MSE perform better. However, because it measures quadratic differences, the MSE is difficult to interpret. Instead, the Mean Absolute Error (MAE) and Mean Absolute Percentage Error (MAPE), which calculate the average absolute gap between predictions and true value, are helpful to measure predictive accuracy (Foster *et al.*, 2021). Therefore, we use accuracy as the main parameter to determine the sufficiency of the model. Accuracy more than 50% is considered sufficient, as explained in the Table S3.3.

Table S6. Classification of accuracy

Accuracy = 100 - MAPE	Accuracy of forecast
Less than 49%	Inaccurate forecast
50% - 79%	Reasonable forecast
80% - 89%	Good forecast
More than 90%	Highly accurate forecast

Source: Kasemset, Sae-Haew and Sopadang (2014)

Table S7. Predicted future retirement ages (unit numbers)

Country	Average unit age as of 2021 (years)	Average unit age as of 2025 (years)	Retire ≤40 years (units)	Retire >40 years (units)	Total units
Argentina	38	0	0	1	1
Australia	34	39	6	53	59
Bangladesh	1	5	14	2	16
Bosnia and Herzegovina	40	32	3	7	10
Brazil	24	34	7	10	17
Bulgaria	43	53	0	31	31
Cambodia	2	6	2	10	12
Canada	37	44	0	22	22
Chile	16	20	9	14	23
China	12	17	3003	10	3013
Colombia	33	32	3	11	14
Croatia	21	25	1	0	1
Czech Republic	47	56	20	47	67
Denmark	27	31	3	0	3
Dominican Republic	20	24	2	3	5
Finland	39	45	1	7	8
France	24	30	0	12	12
Germany	35	38	41	60	101
Greece	27	41	7	0	7
Guatemala	8	12	0	13	13
Honduras	5	9	0	3	3
Hungary	55	0	0	7	7
India	15	19	534	346	880
Indonesia	7	12	74	181	255
Ireland	35	40	3	0	3
Israel	31	38	0	10	10
Italy	32	42	10	8	18
Japan	22	26	50	107	157
Kazakhstan	41	47	0	91	91
Kyrgyzstan	37	29	0	9	9
Laos	6	10	3	0	3
Madagascar	10	14	0	3	3
Malaysia	14	18	17	8	25
Mexico	29	33	0	15	15
Mongolia	23	25	0	12	12
Montenegro	39	0	0	1	1
Morocco	24	34	9	5	14
Myanmar*	12	16	0	3	3
Netherlands	10	14	5	0	5
New Zealand	39	0	0	2	2
North Macedonia	38	41	1	3	4
Pakistan	2	6	0	20	20
Panama	7	11	0	5	5
Peru	22	26	0	1	1
Philippines	9	13	0	60	60

Poland	40	40	5	150	155
Romania	39	42	10	11	21
Russia	44	48	34	272	306
Senegal	5	9	1	1	2
Serbia	46	56	1	16	17
Slovakia	55	0	0	6	6
South Africa	38	49	39	64	103
South Korea	15	20	63	20	83
Spain	37	39	0	11	11
Sri Lanka	8	12	3	0	3
Tajikistan	6	10	0	4	4
Thailand	19	23	4	16	20
Turkey	19	22	53	25	78
Ukraine	54	63	0	93	93
United Arab Emirates*	0	4	4	0	4
United Kingdom	48	42	0	12	12
United States	43	47	26	453	479
Uzbekistan	40	60	7	5	12
Vietnam	9	13	54	25	79
Zambia	5	9	0	3	3
Zimbabwe	24	24	4	5	9

* All units are under construction

Table S8. Predicted future retirement age (capacity)

Country	Average units age as of 2021 (years)	Average units age as of 2025 (years)	Retire ≤40 years (MW)	Retire >40 years (MW)	Total capacity
Argentina	38	0	0	375	375
Australia	34	39	4,160	20,517	24,677
Bangladesh	1	5	8,329	250	8,579
Bosnia and Herzegovina	40	32	830	1,243	2,073
Brazil	24	34	2,503	674	3177
Bulgaria	43	53	0	4,829	4,829
Cambodia	2	6	700	1,020	1,720
Canada	37	44	0	5,679	5679.8
Chile	16	20	2,727	2,214	4,941
China	12	17	1,092,452	1,050	1,093,502
Colombia	33	32	601	1,032	1,633
Croatia	21	25	210	0	210
Czech Republic	47	56	4,630	2,775	7,405
Denmark	27	31	1,180	0	1,180
Dominican Republic	20	24	752	312	1,064
Finland	39	45	39	1,429	1,468
France	24	30	0	3,107	3,107
Germany	35	38	15,481	22,874	38,355
Greece	27	41	2,585	0	2,585
Guatemala	8	12	0	1,010	1,010
Honduras	5	9	0	105	105
Hungary	55	0	0	944	944
India	15	19	229,435	31,965	261400
Indonesia	7	12	36,231	14,594	50,825
Ireland	35	40	915	0	915
Israel	31	38	0	4,900	4,900
Italy	32	42	5,190	1,766	6,956
Japan	22	26	24,678	30,905	55,583
Kazakhstan	41	47	0	11,949	11,949
Kyrgyzstan	37	29	0	910	910
Laos	6	10	1,878	0	1,878
Madagascar	10	14	0	120	120
Malaysia	14	18	12,200	1,080	13,280
Mexico	29	33	0	5,378	5,378
Mongolia	23	25	0	1,010	1,010
Montenegro	39	0	0	225	225
Morocco	24	34	3,792	465	4,257
Myanmar*	12	16	0	160	160
Netherlands	10	14	4,152	0	4,152
New Zealand	39	0	0	500	500
North Macedonia	38	41	225	575	800

Pakistan	2	6	0	8,268	8,268
Panama	7	11	0	426	426
Peru	22	26	0	135	135
Philippines	9	13	0	12,178	12,178
Poland	40	40	4,643	25,636	30,279
Romania	39	42	1,800	2,875	4,675
Russia	44	48	10,068	31,361	41,429
Senegal	5	9	125	30	155
Serbia	46	56	670	3,735	4,405
Slovakia	55	0	0	561	561
South Africa	38	49	27,343	18,465	45,809
South Korea	15	20	40,602	1,692	42,294
Spain	37	39	0	3,089	3,089
Sri Lanka	8	12	900	0	900
Tajikistan	6	10	0	400	400
Thailand	19	23	1,762	4,226	5,988
Turkey	19	22	16,160	4,077	20,238
Ukraine	54	63	0	19,525	19,525
United Arab Emirates	0	4	2,400	0	2,400
United Kingdom	48	42	0	6,328	6,328
United States	43	47	12,381	214,596	226,977
Uzbekistan	40	60	2,100	393	2,493
Vietnam	9	13	26,132	2,225	28,357
Zambia	5	9	0	330	330
Zimbabwe	24	24	1,110	530	1,640

* All units are under construction

Table S9. Remaining lifetime CO_2 emissions

Country	Remaining lifetime CO2 after 2025 (reference scenario)	Remaining lifetime CO2 after 2025 (model prediction)	Discrepancy	Retired units in 2025 (reference scenario)	Retired units in 2025 (model prediction)	Total MW retired in 2025 (reference scenario)	Total MW retired in 2025 (model prediction)
Argentina	0	0	0%	0	1	-	375
Australia	1451.6	578.3	-60%	0	11	-	2,956
Bangladesh	1168.3	1153.8	-1%	0	0	-	-
Bosnia and Herzegovina	53.3	39.9	-25%	0	2	-	600
Brazil	305.5	280.3	-8%	0	3	-	207
Bulgaria	140.8	158.2	12%	2	7	120	802
Cambodia	256.3	296.6	16%	0	0	-	-
Canada	74.1	157.4	112%	0	10	-	2,691
Chile	162.4	524.2	223%	0	6	-	1,172
China	114844.8	49300.9	-57%	20	53	3,070	9,645
Colombia	110.3	107.6	-2%	0	3	-	380
Croatia	16.5	9.9	-40%	0	0	-	-
Czech Republic	307.5	288.2	-6%	0	7	-	868
Denmark	8	31.8	298%	0	2	-	769
Dominican Republic	115.2	119.9	4%	0	1	-	128
Finland	27	32.3	20%	0	3	-	405
France	33	92.7	181%	0	8	-	2,737
Germany	1591.6	1538.6	-3%	3	39	655	10,746
Greece	10.4	132	1169%	0	6	-	1,925
Guatemala	133	163.2	23%	0	0	-	-
Honduras	18.6	24	29%	0	0	-	-
Hungary	5.2	0	-100%	0	3	-	420
India	27150.3	24228.4	-11%	0	56	-	8,664
Indonesia	6095.6	6296.7	3%	0	2	-	800
Ireland	4.8	1.6	-67%	0	0	-	-

Country	Remaining lifetime CO2 after 2025 (reference scenario)	Remaining lifetime CO2 after 2025 (model prediction)	Discrepancy	Retired units in 2025 (reference scenario)	Retired units in 2025 (model prediction)	Total MW retired in 2025 (reference scenario)	Total MW retired in 2025 (model prediction)
Israel	0	184.3	0%	0	10	-	4,900
Italy	31	237.9	667%	0	2	-	336
Japan	3813	3948.6	4%	0	9	-	3,288
Kazakhstan	268.3	400	49%	0	15	-	2,978
Kyrgyzstan	0	68.6	0%	0	9	-	910
Laos	236.6	221	-7%	0	0	-	-
Madagascar	15.6	22.2	42%	0	0	-	-
Malaysia	953.4	1262.5	32%	0	0	-	-
Mexico	216	253.8	18%	0	0	-	-
Mongolia	50.6	67.2	33%	0	3	-	256
Montenegro	0	0	0%	0	1	-	225
Morocco	397.2	331.1	-17%	0	0	-	-
Myanmar*	18.1	24.7	36%	0	0	-	-
Netherlands	65.5	402.7	515%	0	1	-	652
New Zealand	2.8	0	-100%	0	0	-	-
North Macedonia	9.8	1.4	-86%	0	1	-	125
Pakistan	1140.8	1196.1	5%	0	0	-	-
Panama	8.4	75.6	800%	0	3	-	120
Peru	0	14.7	0%	0	1	-	135
Philippines	1403.4	1830	30%	0	2	-	340
Poland	1217.4	1444.9	19%	14	35	2,412	5,749
Romania	30.4	2.1	-93%	0	1	-	330
Russia	1018.7	1213.8	19%	0	36	-	4,620
Senegal	26.8	28.2	5%	0	0	-	-
Serbia	27.8	20	-28%	0	7	-	1,649
Slovakia	1.9	0	-100%	0	2	-	220

Country	Remaining lifetime CO2 after 2025 (reference scenario)	Remaining lifetime CO2 after 2025 (model prediction)	Discrepancy	Retired units in 2025 (reference scenario)	Retired units in 2025 (model prediction)	Total MW retired in 2025 (reference scenario)	Total MW retired in 2025 (model prediction)
South Africa	3454.8	1262.4	-63%	0	5	-	667
South Korea	2744	3823.6	39%	0	2	-	1,120
Spain	4.7	38.5	719%	0	7	-	2,735
Sri Lanka	111.8	99.8	-11%	0	0	-	-
Tajikistan	55	70.2	28%	0	0	-	-
Thailand	354.5	525.6	48%	0	1	-	300
Turkey	1794.4	1597	-11%	0	6	-	1,430
Ukraine	223.4	45.1	-80%	0	9	-	1,730
United Arab Emirates	314.6	286	-9%	0	0	-	-
United Kingdom	10.4	3.4	-67%	1	8	701	4,156
United States	5582.8	4162.8	-25%	0	109	-	47,522
Uzbekistan	63.1	51.7	-18%	0	2	-	600
Vietnam	3544.9	3553	0%	0	3	-	330
Zambia	46.4	50.9	10%	0	0	-	-
Zimbabwe	112.3	116.8	4%	0	3	-	360

Note: Reference scenario assumes a retirement age of 40 years. Model prediction is based on our machine learning prediction.

Table S10. Coal lock-in analysis

Country	Coal lock-in index	Total units in 2025	Total capacity in 2025
Mongolia	6.47	9	0
South Africa	5.22	98	21721
Kazakhstan	5.15	76	8579
India	5.07	824	1473
Indonesia	5.02	253	2970
Poland	5.02	120	4027
Philippines	4.60	58	1720
Bosnia and Herzegovina	4.59	8	2989.2
Cambodia	4.45	12	3769
Vietnam	4.06	76	1083857
Zimbabwe	4.03	6	1253.5
Czech Republic	3.79	60	210
Australia	3.75	48	6537.6
China	3.70	2960	411
Malaysia	3.61	25	936
Madagascar	3.60	3	1063.2
South Korea	3.58	81	370
Bulgaria	3.54	24	27609.4
Laos	3.51	3	660
Morocco	3.48	14	1010.3
Turkey	3.30	72	105
Japan	3.06	148	524
Greece	2.92	1	252736.7
Germany	2.75	62	50025.6
Serbia	2.72	10	915
Sri Lanka	2.71	3	0
United States	2.71	370	6620
Chile	2.65	17	52295.98
Netherlands	2.54	4	8971
Uzbekistan	2.49	10	0
Israel	2.43	0	1878
Thailand	2.43	19	120
Dominican Republic	2.42	4	13280
Russia	2.34	270	5378
Guatemala	2.33	13	754
Pakistan	2.23	20	0
North Macedonia	2.03	3	4257
Zambia	1.93	3	160
Ukraine	1.92	84	3500
Kyrgyzstan	1.75	0	500
Myanmar*	1.70	3	675
Colombia	1.63	11	8268
Denmark	1.50	1	306

Country	Coal lock-in index	Total units in 2025	Total capacity in 2025
Spain	1.47	4	0
Panama	1.41	2	11838
Italy	1.35	16	24530.6
Canada	1.34	12	4345
Croatia	1.28	1	36809.1
Mexico	1.20	15	155
Romania	1.16	20	2756
Brazil	1.10	14	341
Finland	1.04	5	45141.8
Bangladesh	1.03	16	41174
United Kingdom	0.68	4	355
Honduras	0.67	3	900
Tajikistan	0.66	4	400
France	0.66	4	5688
Senegal	0.64	2	18808.16
Ireland	0.62	3	17795
United Arab Emirates	0.61	4	2400
Peru	0.55	0	2172
Argentina	0.00	0	179456.06
Hungary	0.00	4	1893
Montenegro	0.00	0	28027
New Zealand	0.00	2	330
Slovakia	0.00	4	1280
